# Hybrid Supercharged Antibodies: A Rational Approach to Boost Immunoassay Sensitivity via Controlled Nanoparticle Adsorption

**DOI:** 10.1021/acs.langmuir.5c06029

**Published:** 2026-04-03

**Authors:** Junichi Sato, Keisuke Kasahara, Satoru Nagatoishi, Keisuke Murakami, Daisuke Kuroda, Jose M. M. Caaveiro, Hirokazu Nagai, Kouhei Tsumoto

**Affiliations:** † Department of Chemistry and Biotechnology, Graduate School of Engineering, The University of Tokyo, 7-3-1, Hongo, Bunkyo-ku, Tokyo 113-8656, Japan; ‡ Biomaterial Business Development Department, Asahi Kasei Corporation, Hibiya Mitsui Tower 1-1-2 Yurakucho, Chiyoda-ku, Tokyo 100-0006, Japan; § Department of Bioengineering, Graduate School of Engineering, The University of Tokyo, 7-3-1, Hongo, Bunkyo-ku, Tokyo 113-8656, Japan; ∥ Laboratory of Protein Drug Discovery, Graduate School of Pharmaceutical Sciences, Kyushu University, 3-1-1 Maidashi, Higashi-ku, Fukuoka 812-8582, Japan; ⊥ Medical Device Development and Regulation Research Center, School of Engineering, The University of Tokyo, 7-3-1 Hongo, Bunkyo-ku, Tokyo 113-8656, Japan; # Department of Biosciences, College of Humanities and Sciences, Nihon University, 3-25-40 Sakurajosui Setagaya-Ku, Tokyo 156-8550, Japan

## Abstract

This study presents a novel approach to enhance the sensitivity of a lateral flow immunoassay by computationally designing supercharged antibodies that optimize both the adsorption amount and molecular orientation on nanoparticle surfaces. We engineered immunoglobulin G antibodies with positively charged Fc domains and negatively charged Fab domains to create charge-polarized molecules for controlled interaction with negatively charged cellulose nanoparticles (NanoAct). The supercharged antibodies retained physicochemical properties and antigen-binding affinities identical to those of the wild-type antibody. Quantitative analysis showed that positively supercharged Fc domains enhanced antibody adsorption, and the charge-polarized design (featuring a negatively charged Fab and a positively charged Fc; c−10/Fc-pos14) enhanced relative Fab accessibility on the nanoparticle surface. Interaction analyses between supercharged antibodies and NanoAct using isothermal titration calorimetry quantitatively revealed that the c−10/Fc-pos14 antibody adsorbed onto NanoAct in the tail-on orientation. Consequently, lateral flow immunoassay performance tests demonstrated an 8-fold improvement in the limit of detection from 25 to 3.13 ng/mL, without increasing nonspecific binding. The key design principle involves maintaining sufficient charge separation between the domains to ensure proper orientation control. This supercharging approach represents a promising strategy for boosting immunoassay sensitivity while preserving sufficiently low noise levels with potential applications in other antibody-based diagnostic platforms.

## Graphical abstract

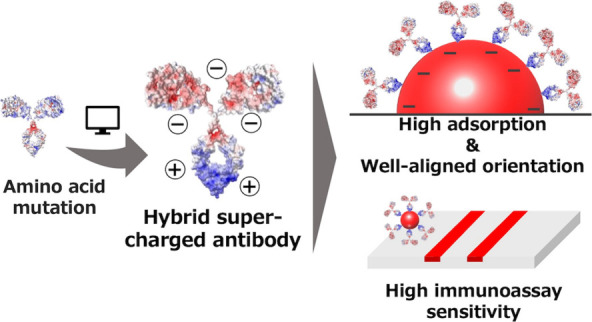

## Introduction

The lateral flow immunoassay (LFIA) is a highly versatile diagnostic tool. They have also proven useful in the recent emerging infectious diseases field,^1^ creating significant societal demand. Nearly half a century has passed since the concept of LFIA was first proposed, and there have been extensive developments in techniques and methods to improve its sensitivity.^2,3^ The core detection reagents in modern LFIA combine nanoparticles and antibodies. Nanoparticles can be visually identified based on their detection signals, whereas antibodies offer high specificity for capturing target antigens. To further boost detection sensitivity using nanoparticles and antibodies, various techniques have been proposed for immobilizing antibodies on nanoparticles. These immobilization techniques can be broadly categorized into three main types: specific tagging, covalent bonding, and physical adsorption.^4–6^ Among these, physical adsorption is a low-cost, simple method with high reproducibility that is used in many commercially available LFIAs. The essence of the aforementioned immobilization techniques is to either enhance adsorption capacity or improve antibody orientation. The pH change technique is a versatile and simple method to achieve these goals.^4–6^ However, most approaches only improve the antibody orientation.^7,8^ Although some studies have reported improvements in sensitivity through better orientation,^9^ the immobilization method still relies on covalent bonding. Thus, an ideal method that simultaneously improves both adsorption and orientation through the physical adsorption of antibodies onto nanoparticles has yet to be developed.

In this study, we designed a supercharged antibody by modifying its surface charge through amino acid mutations to enhance the amount of antibody adsorption and to improve antibody orientation, thereby increasing sensitivity (Figure 1). Through this approach, we aimed to boost detection sensitivity by increasing the amount of physically adsorbed antibodies and favoring their tail-on orientation on the nanoparticle surface.

**Figure 1:**
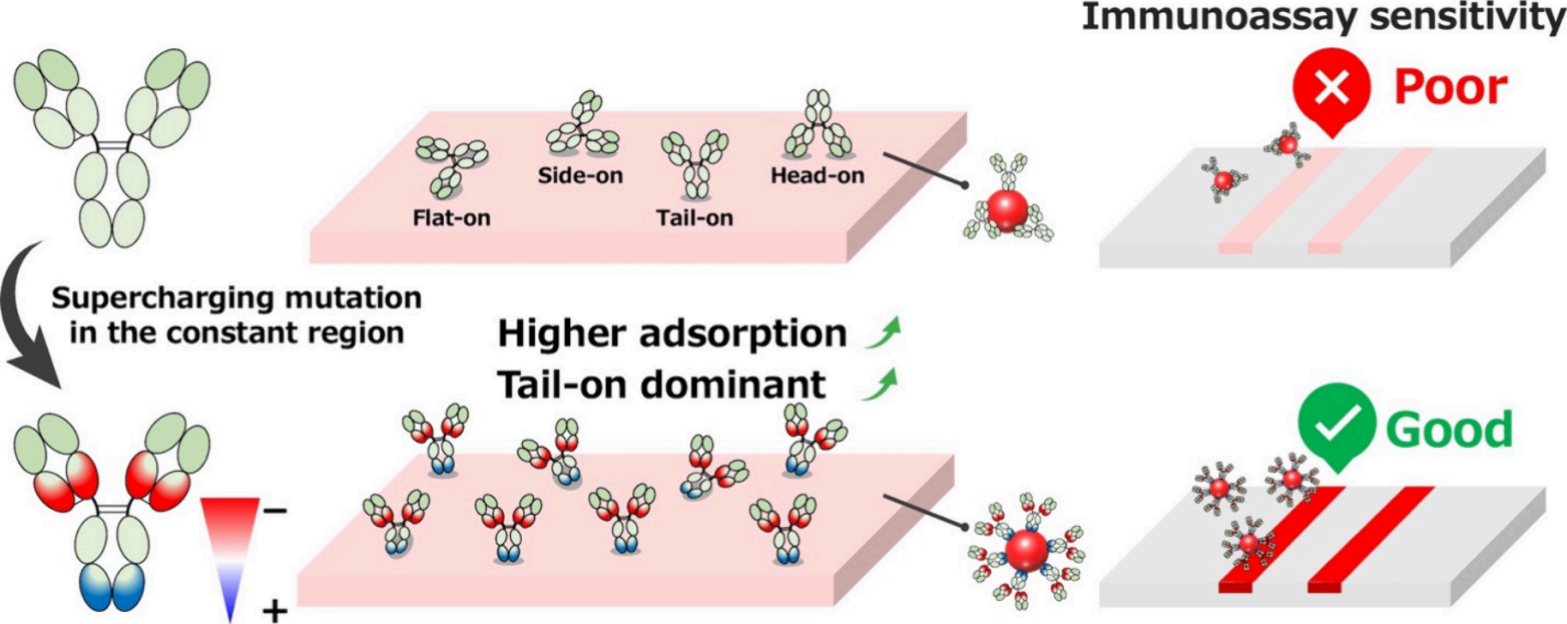
Conceptual diagram illustrating the supercharging of IgG antibodies to enhance adsorption and control molecular orientation on nanoparticles, thereby improving analytical sensitivity in LFIA.

## Results and Discussion

We adopted supercharged antibodies to enhance antibody adsorption and improve antibody orientation (Figure 1). Lawrence et al. first proposed a supercharging technique for protein surfaces to enhance their thermostability.^10^ Miklos et al. have reported the supercharging of antibodies using computational science.^11,12^ Recently, we employed computational technology to design supercharged antibodies that introduce charged amino acid residues onto the antibody surface without destabilizing its physical properties.^13,14^ Furthermore, we demonstrated the electrostatic-force-driven physical adsorption of antibodies onto negatively charged cellulose nanoparticles, whereby the positive charge of the antibody promoted effective interaction with the nanoparticle surface.^15^ Based on our previous studies, we attempted to design a hybrid surface-charged antibody with both positively and negatively charged sites within a single antibody molecule by supercharging; the Fc domain of an immunoglobulin G (IgG)-type antibody was supercharged with a positive charge and the Fab domain with a negative charge (Figure 1). In this hybrid surface-charged antibody, the Fc moiety, which is irrelevant to antigen binding, was expected to face the nanoparticle surface, thereby promoting tail-on adsorption. Conversely, the Fab moiety, which is critical for antigen binding, is expected to face outward, thereby repelling the nanoparticle. Moreover, because the Fc region carries an excessively positive charge that cannot occur naturally, enhanced adsorption onto the nanoparticles is expected.

### Computational Design and Preparation of IgGs

To create a universal antibody that can adsorb onto negatively charged cellulose nanoparticles with the Fab facing outward, the Fc domain must always have a higher isoelectric point (pI) than the Fab domain (Figure 1). However, the pI of the Fc in human IgG1, computed using PDB2PQR,^16–18^ was 7.6 (Table S1), and the pI of the Fab varied significantly depending on the Fv sequence. In this study, we used the 3C1 antibody, which has a relatively high pI, as a model for the variable region.^19^ This antibody, derived from mouse IgG1κ, was used to create a chimeric antibody with a constant region sequence from human IgG1κ, referred to as h3C1. In practice, the h3C1 Fab has a high pI of 9.0 (Table S1). To design a constant region that is positively charged on the Fc side and negatively charged on the Fab side, regardless of the Fv sequence and under universal conditions, we performed independent computational supercharging designs for both the Fc and Fab regions using the previously described method.^13,14^

Supercharging of the Fc domain, restricted to introducing mutations only in the C_H_3 domain (six mutations; E345_H_R, Q386_H_K, N389_H_K, Q419_H_K, H433_H_K, Q438_H_K), generated a positively supercharged Fc with a net charge of +14 (Fc-pos14) compared with the wild-type (WT), which was zero based on the crystal structure (PDB ID: 4W4N)^20^ (Figures 2a and S1). Subsequently, starting from the crystal structure of a human IgG1κ Fab antibody (PDB: 5WUV),^21^ our design in the C_H_1 and C_L_ regions indicated the seven mutations (K133_H_D, S160_H_D, S165_H_E, S191_H_D, K205_H_E, K145_L_E, Q199_L_E) that decrease the net charge by 10 (c−10) (Figures 2b and S1). Together, these mutations shifted the surface charge of the Fc and Fab domains, increasing the pI of Fc from 7.6 to 9.7 and decreasing the pI of Fab from 9.0 to 6.5 (Table S1).

**Figure 2:**
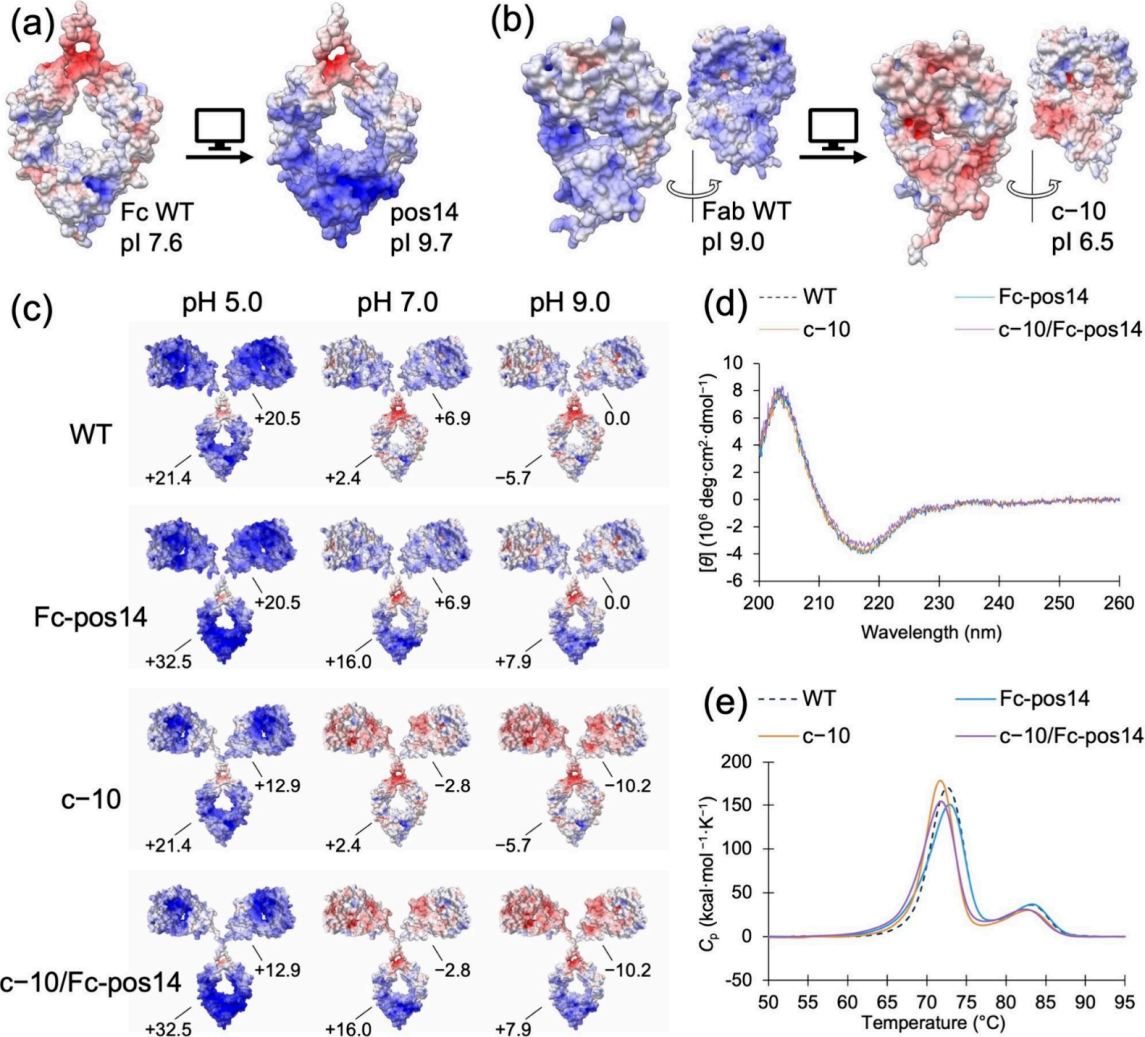
Computational design of supercharged IgGs and physicochemical analyses. Surface representations of (a) human IgG1 Fc WT and pos14 mutant and (b) h3C1 Fab WT and c−10 mutant, colored according to electrostatic potential at pH 7.0. (c) Surface representations and the net charge of the Fab and Fc domains at pH 5.0, 7.0, and 9.0. The pIs, electrostatic potentials, and net charge were computed with PDB2PQR^16,17^ and APBS.^22^ The scale ranges from −10 *kT*/e (red) to 10 *kT*/e (blue). (d) CD spectra and (e) differential scanning calorimetry profiles of the h3C1 IgGs in PBS.

The electrostatic potentials of h3C1 IgGs at pH 5.0, 7.0, and 9.0 were computed using PDB2PQR and APBS from the structure modeled by MODELLER (Figure 2c).^16–18,22,23^ At pH 5.0, the entire surface of the four IgGs was positively charged because of protonation of the histidine residues. As the pH increased, the differences in the surface charge became apparent. Except in the WT, the net charge of each Fc domain exceeded that of its Fab domain. Mutations in Fc-pos14 caused the Fc domain to acquire a positive net charge, even at pH 9.0. Similarly, c−10 mutations also conferred a negative net charge on the Fab domain.

We cloned the DNA plasmids of the heavy and light chains of the four types of h3C1 IgGs with two variations each for Fc and Fab (WT, Fc-pos14, c−10, and c−10/Fc-pos14), followed by expression by ExpiCHO cells. All four IgGs were purified as monomers with a single major peak, although c−10 mutations decreased yields (Figure S2). We confirmed that these h3C1 IgG mutants had physicochemical properties identical to those of WT. Circular dichroism (CD) spectra and the midpoint temperature of denaturation (*T*_m_) were perfectly preserved, indicating that the main-chain structures were not affected by the mutations (Figure 2d,e, and Table S2). Surface plasmon resonance (SPR) analysis indicated that the binding affinity and kinetic parameters of the IgGs to the antigen were identical (Figure S3 and Table S3).

### Quantification of Antibody Adsorption on Cellulose Nanoparticles

The amount of antibody adsorbed onto the cellulose nanoparticles (NanoAct) was quantified using a bicinchoninic acid (BCA) assay before and after the washing step during the conjugation process (Figures S4 and S5) to characterize the nanoparticle–antibody complexes used for LFIA. Before the washing step, all added antibodies were adsorbed onto NanoAct except for the following three conditions: c−10 at pH 7.0 and 9.0, and WT at pH 9.0 (Figure S4c).

Conversely, the amount of antibody adsorbed after the washing step was correlated with the net charge of the antibodies (Figure 3a). Fc-pos14 exhibited a high level of antibody adsorption across all pH values, making it the most effective antibody for increasing retention on the particles. This is because Fc-pos14 maintains a positive charge across a wide pH range of 5.0–9.0. In contrast, c−10/Fc-pos14 generally exhibited reduced antibody adsorption compared with Fc-pos14 across all pH levels, likely due to the negative supercharging introduced into the Fab region of the IgG. Indeed, c−10, which is negatively supercharged only in the Fab region, showed lower antibody adsorption than other antibodies across all pH values. Specifically, when c−10 was conjugated to NanoAct at pH 9.0, less than 10% of the added antibodies adsorbed onto NanoAct. Additionally, both Fc-pos14 and c−10/Fc-pos14 displayed antibody adsorption levels equal to or greater than those of WT across a broad pH range (pH 5.0–9.0). They maintained higher adsorption levels than the WT even under alkaline pH conditions. These findings suggest that the positive supercharging of the Fc region not only enhances antibody adsorption onto NanoAct but also suppresses antibody desorption from NanoAct.

**Figure 3:**
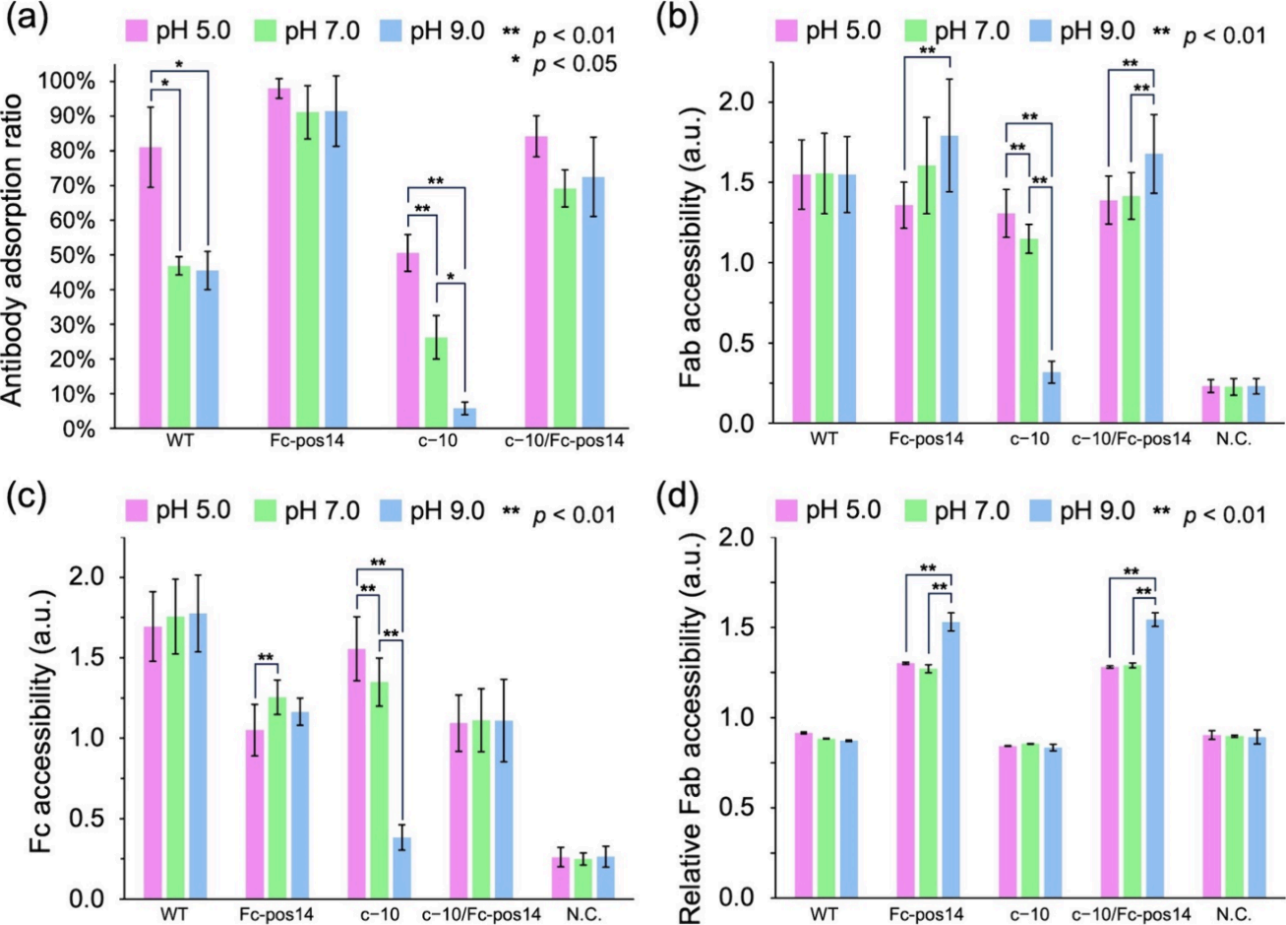
Evaluation of antibody adsorption and Fab/Fc accessibility. (a) The antibody adsorption ratio measured after the washing step in the conjugation process. The amount of antibody fed in the physical adsorption step (see Figure S5) is defined as 100%. A significance test was conducted between different pH conditions for each antibody but not conducted between different antibodies. (b) Fab accessibility, (c) Fc accessibility, and (d) relative Fab accessibility of h3C1 IgGs conjugated onto NanoAct at different pH. D-PBS (−) was used as a negative control (N.C.).

### Evaluation of Fab and Fc Accessibility of Antibodies on Cellulose Nanoparticles

For further characterization of the nanoparticle–antibody complexes used in LFIA, we investigated the accessibility of the Fab and Fc regions of the NanoAct-labeled antibodies using Fab-specific or Fc-specific anti-human IgG antibodies. While various methods have been previously reported to evaluate the accessibility of antibodies bound to nanoparticles,^9,24^ we quantified accessibility using an enzymatic assay with horseradish peroxidase (HRP)-labeled anti-human IgG and 3,3′,5,5′-tetramethylbenzidine (Figure S6). During method development, we also tested more functional approaches, including an HRP-labeled antigen assay and ITC with antigen–nanoparticle conjugates; however, these proved impractical due to persistent nonspecific coloration and undetectable heat signals, respectively. Therefore, we adopted the anti-Fab/anti-Fc enzyme assay, which directly indicates whether adsorbed antibodies are preferentially Fab- or Fc-exposed under each condition. Herein, we quantified Fab and Fc accessibility when the amount of NanoAct was constant. The results showed that both Fab and Fc accessibility of the WT remained almost constant across all pH levels (Figure 3b,c). This appears to contradict earlier findings regarding antibody amounts. Specifically, it is challenging to explain why the Fab and Fc accessibility did not change despite more antibodies being adsorbed onto NanoAct in pH 5.0 buffer compared with higher pH solutions. These results can be interpreted as follows: at pH 5.0, antibodies are densely packed on NanoAct surfaces, allowing small molecules like BCA to access and be detected as adsorbed antibodies in the BCA assay. Conversely, because anti-human IgG antibodies are large molecules, comparable in size to WT molecules, some of the Fab and Fc regions of WT molecules may be inaccessible to these anti-human IgG antibodies. At pH 7.0 and 9.0, WT molecules are spaced sufficiently apart on NanoAct surfaces, allowing HRP-labeled anti-human IgG antibodies to access these WT molecules. Assuming that NanoAct is a perfect sphere with a smooth surface, the maximum number of IgG molecules that can form a monolayer on its surface is approximately 1.0 × 10^4^. In this experiment, the ratio of the added antibody molecules to NanoAct particles was roughly 1.2 × 10^4^ (see Experimental Details in Supporting Information), suggesting that when all added antibodies are adsorbed, NanoAct surfaces become “crowded.”

Next, the Fab accessibility of c−10 varied significantly with the pH of the buffer solution during the conjugation process, and similar results were observed for Fc accessibility. This indicates that the differences in Fab and Fc accessibility of c−10 due to pH shifts mainly result from variations in the amount of antibody adsorbed onto NanoAct. Therefore, by calculating the ratio of Fab to Fc accessibility, we defined a new parameter called “relative Fab accessibility,” which eliminates the influence of the total amount of antibody adsorbed onto NanoAct. The relative Fab accessibility of c−10 remained constant across all pH conditions during the conjugation process (Figure 3d).

Finally, the Fc-positively supercharged antibodies (Fc-pos14 and c−10/Fc-pos14) showed significantly improved Fab accessibility when conjugated to NanoAct at pH 9.0 (Figure 3b). In contrast, significant differences in Fc accessibility were only observed when Fc-pos14 was conjugated to NanoAct at lower pH levels (Figure 3c). Accordingly, relative Fab accessibility was greatly enhanced for the two positively supercharged antibodies conjugated at pH 9.0 (Figure 3d). This improvement is likely due to the positive supercharging of the Fc region and the pH control during the conjugation process, which results in only the Fc region being positively charged and favorably oriented to bind to the negatively charged NanoAct. In summary, these findings strongly suggest that the antibody orientation on the nanoparticles can be controlled using the supercharging technique.

### Adsorption and Orientation Analysis of Antibodies in Isothermal Titration Calorimetry (ITC)

To evaluate the adsorption behavior of the four types of antibodies onto NanoAct in detail, we performed ITC to analyze the interaction between NanoAct and the antibodies (Figures 4a–d, S7, Tables 1, and S4–S6) following our previous study.^15^ Simultaneously, the amount of antibody remaining in the supernatant after the binding reaction in the ITC cell was calculated from the UV absorbance, enabling us to calculate the molar ratio of adsorption, including nonthermal reactions, which we refer to as *N*_sup_ in this study. The *N*_sup_ values exceeded the binding molar ratio (*N*) derived from the heat absorption or release curves of the ITC measurements (Tables 1, S5, and S6). This indicates that the observed exothermic or endothermic reactions may represent the initial phase of antibody adsorption onto NanoAct. Therefore, the ITC results provide direct evidence of the adsorption properties of the antibodies.

**Figure 4:**
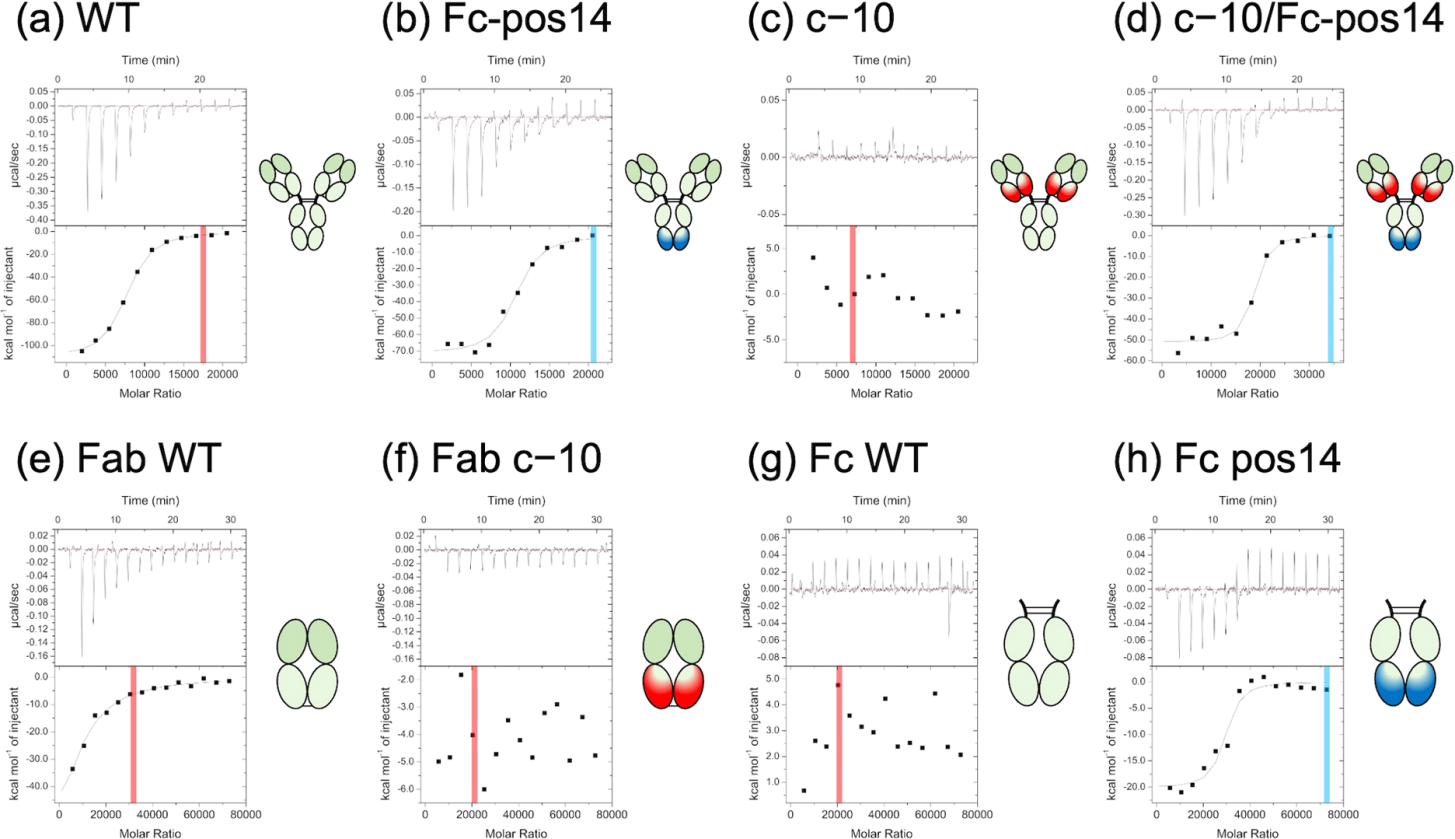
ITC analyses of the interaction between NanoAct particles and IgGs or their fragments at pH 9.0. (a) IgG WT, (b) IgG Fc-pos14, (c) IgG c−10, (d) IgG c−10/Fc-pos14, (e) Fab domain WT, (f) Fab domain c−10, (g) Fc domain WT, and (h) Fc domain pos14. Red or blue lines indicate the average *N*_sup_ in the measurements, with red representing unsaturated and blue representing saturated conditions.

**Table 1:** Molar Ratio and Affinity of the Interaction between h3C1 IgGs or Their Domains and NanoAct at pH 9.0^a^

		*N*	*N*_sup_	*K*_D_ (nM)
IgG	WT	7300 ± 300	17,500 ± 700	45.2 ± 12.6
	Fc-pos14	9600 ± 1200	>20,500	52.0 ± 20.1
	c−10	N.D.^b^	7200 ± 600	N.D.^b^
	c−10/Fc-pos14	17,100 ± 600	>34,000	13.5 ± 0.3
Fab	WT	N.D.^c^	30,600 ± 14,900	906 ± 584
	c−10	N.D.^b^	18,900 ± 2800	N.D.^b^
Fc	WT	N.D.^b^	21,000 ± 1300	N.D.^b^
	pos14	28,000 ± 400	>72,800	12.5 ± 11.1

a Values are mean ± S.D. of three independent experiments.

b Not determined. The reaction curve could not be obtained.

c Not determined. The affinity was weak, and the *N* value was inaccurate.

Our previous studies indicated that the reaction was endothermic under acidic conditions and became more exothermic as the pH approached basic conditions, with a transition occurring at approximately pH 5.5.^15^ In this study, small endothermic reactions were observed at pH 5.0 (Figure S7b–d), suggesting that the transition pH between endothermic and exothermic reactions was also slightly higher than pH 5.0. Therefore, it was difficult to confirm the thermodynamic parameters (Table S5). Moreover, for an antibody with a relatively low pI, increasing pH caused the surface charge to become more negative, making it harder for the antibody to approach and adsorb onto NanoAct^15^ This pH-dependent effect was also observed in this study, especially for WT and c−10. Almost all antibodies showed thermal reactions and were fully adsorbed onto the particles (*N*_sup_ > 20,500), suggesting saturation. In other cases, the thermal reactions were absent, and the adsorption amount followed the order: WT at pH 9.0 > c−10 at pH 7.0 > c−10 at pH 9.0, and the thermodynamic parameters in c−10 at pH 9.0 could not be obtained (Tables 1, S4, and S6). These findings correlated with the quantification of antibody adsorption on NanoAct before the washing step (Figure S4c). The Fab and Fc domains of WT at pH 9.0 were estimated to have a net charge of 0.0 and −5.7, respectively, and the corresponding values of c−10 should be −2.8 and +2.4 at pH 7.0 and −10.2 and −5.7 at pH 9.0, respectively (Figure 2c). These results suggest that the antibody domains require a positive net charge to efficiently adsorb onto the particles. Positive supercharging of the Fc domain helped to recover IgG adsorption at higher pH levels. The adsorption of both Fc-pos14 and c−10/Fc-pos14 reached saturation under all conditions. The *N* value for c−10/Fc-pos14 at pH 9.0 (17,100 ± 600) was significantly higher than all the other results (11,400 or less) (Tables 1, S5, and S6). To confirm which domains generate thermal reactions during adsorption, we conducted ITC measurements with the Fab and Fc domains at pH 9.0 (Figure 4e–h, Tables 1, and S4). At pH 9.0, only the Fc domain pos14 and the Fab domain WT indicated exothermic reactions, while the Fc domain WT and Fab domain c−10 did not. As previously suggested, a positively charged surface is important for adsorption and thermal reactions. Based on these results, most exothermic reactions of c−10/Fc-pos14 at pH 9.0 probably originate from the interaction between NanoAct and the Fc domain, continuing longer than the other IgGs’ adsorption (Figure 4d,f,h). This provides strong indirect evidence that c−10/Fc-pos14 preferentially adsorbs onto the particles in a tail-on orientation. Regarding WT at pH 9.0, the Fab domain adsorbs more easily than the Fc domain, leading to head-on orientation and deactivation of antibodies. The exothermic reactions of Fc-pos14 at pH 9.0 converged earlier with an *N* value of 9600 (Figure 4b and Table 1). The WT Fab domain had a weaker affinity for the particles than the Fc domain pos14. However, it might be sufficient to facilitate the adsorption of Fc-pos14 IgG in various molecular orientations (including side-on and flat-on) and fill NanoAct surfaces earlier than c−10/Fc-pos14.

### Improvement in the Limit of Detection of LFIA

To evaluate the effects of enhanced antibody adsorption and improved molecular orientation on LFIA, test strips were prepared according to a previously reported protocol^15^ (Figures S8 and S9). The limit of detection (LOD) was evaluated using a dilution series of the recombinant antigen (SARS-CoV-2 spike protein). The color intensity of the test strips was quantified using an optical reader. The LOD was defined as the lowest antigen concentration that produced a color intensity exceeding a preset threshold corresponding to the level at which the color was barely visible to the naked eye.

As shown in Figure 5a, the LOD for the WT was lowest when the antibody was conjugated at pH 5.0 and 7.0, with a value of 25 ng/mL. In contrast, the LOD for Fc-pos14 and c−10/Fc-pos14 improved to 6.25 and 3.13 ng/mL, respectively, when the conjugation was performed at pH 9.0. These results indicate that supercharging can enhance the LOD of LFIA by up to 8-fold. The fact that c−10/Fc-pos14 and Fc-pos14 supercharged antibodies showed the best LOD when conjugated at pH 9.0 is consistent with the result that relative Fab accessibility was highest at this pH, as shown in Figure 3d.

**Figure 5:**
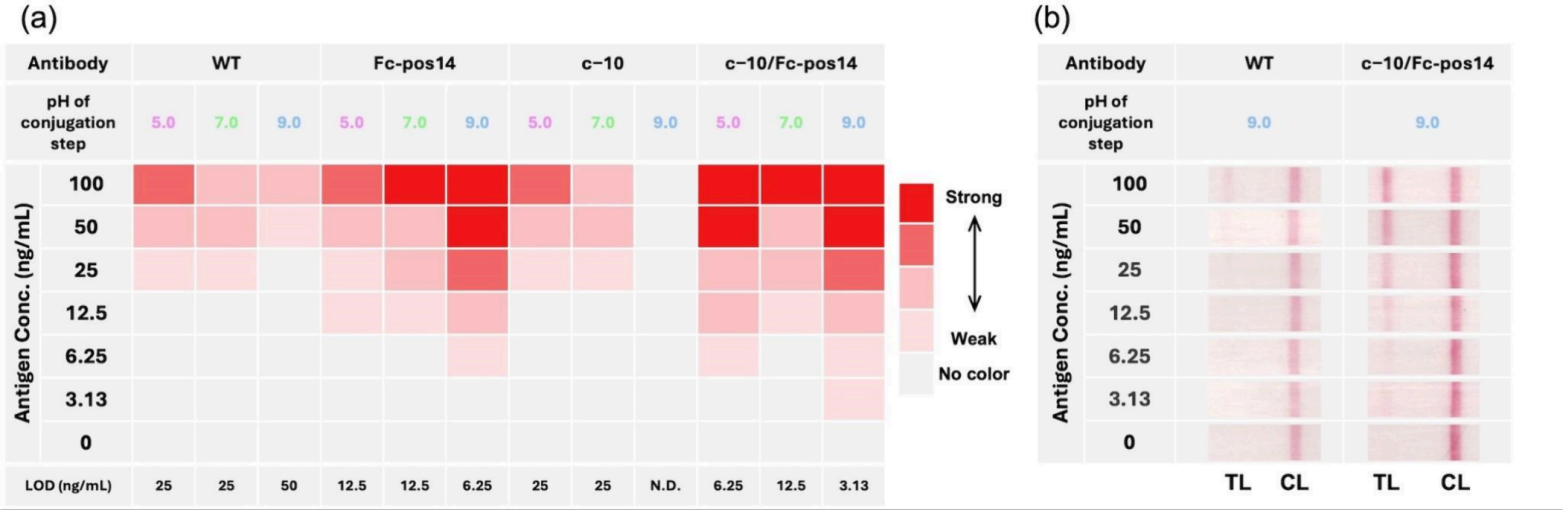
Comparative analysis of immunodetection sensitivity for SARS-CoV-2 spike protein across varying antigen concentrations and antibody adsorption conditions. (a) The limit of detection of the LFIA using NanoAct conjugated with each antibody at different pH levels. Each cell in the table is shaded red with an intensity that correlates with the color intensity of the test line. (b) Representative photographs of LFIA test strips after immunodetection. “TL” and “CL” in the figure represent the positions of the test and control lines, respectively. All measured color intensities and related photographic data are provided in Figure S10.

Additionally, for WT, the color intensity at the same antigen concentration decreased as the conjugation pH increased, whereas for Fc-pos14, the color intensity increased with higher conjugation pH. The former suggests that having a high positive charge on the antibody can be advantageous when conjugating antibodies to NanoAct for use in LFIA, as discussed in the previous study.^15^ However, the latter implies that it is more important for the antibody molecule to have an appropriate charge distribution; the charge states of the Fc and Fab regions are more crucial than the overall charge state. This finding is further supported by the observation that c−10/Fc-pos14 exhibited stronger color intensity than Fc-pos14 across all pH levels in the conjugation process and antigen concentrations in LFIA (Figure S10). In other words, c−10/Fc-pos14, with its negatively supercharged Fab region, was more “polarized” under all pH conditions than Fc-pos14, allowing the Fc region to preferentially adsorb onto NanoAct.

In contrast, c−10/Fc-pos14 did not show a monotonic trend like Fc-pos14 but instead exhibited minimum color intensity when conjugated to NanoAct at pH 7.0. This behavior can be reasonably interpreted as a combination of the behavior of Fc-pos14 and c−10. Specifically, as the conjugation pH increased, the color intensity of Fc-pos14 increased monotonically, whereas that of c−10 decreased monotonically. Consequently, c−10/Fc-pos14 showed a biphasic trend: a decrease in color intensity from pH 5.0 to 7.0, resembling the behavior of c−10, followed by an increase from pH 7.0 to 9.0, similar to Fc-pos14. In other words, the lower sensitivity observed at pH 7.0 does not represent an absolute decrease; rather, it corresponds to a relative minimum arising from enhanced sensitivity at pH 5.0 and pH 9.0. Thus, it is reasonable to conclude that c−10/Fc-pos14 possesses characteristics of both Fc-pos14 and c−10. While c−10 exhibited an LOD comparable to that of WT when conjugated at pH 5.0 and 7.0, it failed to detect even 100 ng/mL of antigen when conjugated at pH 9.0. This was because the amount of antibody on NanoAct surfaces was extremely low, preventing the capture of antigen in positive specimens. Indeed, the test strips prepared with c−10 conjugated to NanoAct at pH 9.0 showed significantly weaker color intensity in the control line than other test strips (Figure S10b). This indicated that the amount of detection antibody captured by the anti-human IgG antibody on the control line was very low, in agreement with the antibody adsorption measurements and ITC analysis. Accordingly, we quantified color intensities of the control lines in Figure S10b using the optical reader and averaged them per conjugation pH. We then correlated these averaged intensities with the postwash antibody adsorption ratios (see Figure 3a). As shown in Figure S11, control-line intensity positively correlates with adsorption (*R* ^2^ = 0.66), with both metrics lowest for c−10, particularly when conjugated at pH 9.0. These results reinforce the quantitative integrity of the conjugates and the functional validation of the immunoassay.

An additional notable observation was that none of the supercharged antibodies exhibited significant nonspecific coloration when negative specimens (0 ng/mL antigen) were used. Nonspecific coloration is the most concerning and common issue in practical LFIA applications. However, no intensification of nonspecific coloration due to supercharging was observed, and no problematic background noise occurred. Generally, efforts to optimize LFIA device configurations to improve sensitivity tend to exacerbate nonspecific coloration, thereby compromising the signal-to-noise ratio. Nevertheless, as demonstrated above, supercharging appears to be an optimal approach for boosting the LFIA sensitivity because it enhances the signal intensity without introducing additional noise.

## Conclusions

This study introduces a novel approach to boost the sensitivity of an LFIA through the rational design of supercharged antibodies. By considering the intrinsic negative charge of the cellulose nanoparticles, we computationally engineered the constant regions of an IgG antibody, in which we independently modifying the positively charged Fc domain (Fc-pos14) and the negatively charged Fab domain (c−10), while preserving their structural and functional integrity. This engineered antibody, with overall charge polarization, demonstrated improved adsorption and controlled molecular orientation, leading to an 8-fold improvement in the limit of detection of LFIA. Unlike conventional strategies for improving LFIA performance, which often suffer from suboptimal antigen orientation, limited adsorption capacity, increased nonspecific binding, or reliance on complex chemical modifications,^25,26^ our approach achieved excellent signal-to-noise ratios without amplifying background signals, relying solely on simple physical adsorption.

The key design principle is to maintain sufficient charge separation between domains, approximately 18 charge units in our case (Fc: +7.9 and Fab: −10.2 at pH 9.0), to ensure proper orientation control. The positively charged Fc domain promotes robust adsorption onto the negatively charged nanoparticle surface, whereas the negatively charged Fab domain is repelled from the surface and oriented outward, thereby maximizing target recognition. Remarkably, the best-performing antibody (c−10/Fc-pos14) had an overall negative net charge (−12.5 at pH 9.0), yet achieved superior performance, highlighting the significance of domain-specific charge engineering over global charge modification. Moreover, the charge-separation design principle can be extended to antibodies with diverse isoelectric profiles. Since this study employed a model antibody with a relatively high Fab pI (9.0), for instance, those with naturally lower Fv pI values may require less negative supercharging in their Fab region than our c−10 mutant.

Our findings demonstrate how the supercharging of antibodies can unlock significant improvements in immunoassay technologies. We believe that the proposed technique provides an innovative direction for antibody design, not only to improve LFIA sensitivity but also to advance other antibody-based assays involving electrostatic interfaces. Our previous research suggests that polar molecules such as c−10/Fc-pos14 are prone to aggregation under low-salt conditions.^14^ Practically, we purified and stored the antibodies in a high-salt concentration buffer to prevent aggregation. The supercharging design presented herein offers the potential for broader applications, including immunoprecipitation, biosensing, and therapeutic delivery systems.

## Methods

### Computational Supercharging Design

The Fc domain of human IgG1 with a net charge of +14 (Fc-pos14) and the constant regions in the human IgG1κ Fab domain with a net charge decreased by 10 (c−10) were designed using the same method as previously described.^13^ The initial structure of Fc was obtained from the PDB files of its crystal structure (ID: 4W4N^20^). As the initial structure of the Fab, the crystal structure of the certolizumab Fab was selected because it has the same sequence as the human IgG1κ constant regions (ID: 5WUV^21^). These structures were refined using the Rosetta force field (REF2015) through a relaxed application with all-atom constraints.^27,28^ Based on the relaxed structure, a computational design was subsequently developed with a supercharge application.^12^ Residue files were prepared to limit the designable regions to the C_H_3 domain of Fc and the C_H_1 and C_L_ domains of Fab. The target net charge for the Fc was set to +10 compared with the WT’s net charge of zero, and that for the Fab was set to −7 compared with the certolizumab net charge of +4. The suggested mutations in Fc for each heavy chain (E345R, Q419K, H433K, and Q438K in one chain and E345R, Q386K, N389K, Q419K, and H433K in the other chain) were merged (Figure S1).

### Modeling Structures and Surface Representation of the Electrostatic Potentials

The model structure of h3C1 Fab was constructed using the 3C1 Fv and certolizumab constant regions extracted from the PDB files (IDs: 7DCC for 3C1 ^19^ and 5WUV for certolizumab). Residues not present in the crystal structure and model structures of the mutants were modeled using MODELLER 10.4.^23^ The pI and electrostatic potential of the folded protein structures were calculated using PDB2PQR^16,17^ and APBS^22^ via a web server that supported both methods.^18^ All figures of protein structures were generated using the UCSF Chimera.^29^

### Preparation of the IgGs as Recombinant Proteins

Recombinant h3C1 IgGs were expressed in the ExpiCHO expression system using the max titer protocol (Thermo Fisher Scientific, USA) as previously described.^13^ Briefly, gene fragments encoding the heavy or light chains of the h3C1 IgG were cloned and inserted into pcDNA3.4 vectors (Thermo Fisher Scientific). ExpiCHO cells were transfected with the expression vector, and the supernatant was harvested after 14 days of culture at 32 °C with 5% CO_2_. The culture supernatant was collected via centrifugation for 10 min at 5000 × *g* and filtered through 0.8 μm filters (Advantec, Japan). It was loaded onto 1 mL of rProtein A Sepharose Fast Flow resin (Cytiva, Japan) equilibrated with 1× phosphate-buffered saline (PBS), followed by washing with 10 mL of 1× PBS. An additional wash with IgGs containing Fc-pos14 mutations was performed using 5 mL of 20 mM Tris–HCl (pH 8.0), 200 mM arginine–HCl, and 500 mM NaCl. IgGs were eluted with 4 mL of Pierce IgG elution buffer (Thermo Fisher Scientific), and the eluted fractions were immediately neutralized by adding 0.4 mL of 0.5 M Tris, 2 M NaCl, and 400 mM arginine–HCl (pH ∼ 9). The eluate was then dialyzed in 20 mM Tris–HCl (pH 8.0), 200 mM arginine–HCl, and 500 mM NaCl overnight, followed by further purification via size-exclusion chromatography (SEC) using a HiLoad 16/600 Superdex 200 pg column (Cytiva) equilibrated with the same buffer at 4 °C. The purified samples were stored at 4 °C in the same buffer, and the buffer was changed just before using them in the following assay.

### Preparation of the Fab and Fc Domains as Recombinant Proteins

Recombinant human IgG1 Fc was expressed in the ExpiCHO expression system and purified using rProtein A Sepharose Fast Flow resin as described above. Recombinant h3C1 Fabs were obtained via papain digestion of IgGs using a Pierce Fab Preparation Kit (Thermo Fisher Scientific) following the manufacturer’s protocol.

Both Fcs and Fabs were further purified using SEC with a HiLoad 16/600 Superdex 75 pg column (Cytiva) equilibrated with 20 mM Tris–HCl (pH 8.0), 200 mM arginine–HCl, and 500 mM NaCl for Fc domain pos14 and 1× PBS for other proteins at 4 °C.

### Preparation of the Antigen (RBD) as Recombinant Proteins

The DNA sequence encoding the SARS-CoV-2 Spike RBD was codon-optimized and synthesized by Integrated DNA Technologies (USA). It was subcloned into the pcDNA3.4 vectors (Thermo Fisher Scientific) with a His_6_ tag fused to the C-terminus. The RBD was expressed in Expi293 cells (Thermo Fisher Scientific) following the manufacturer’s standard protocol. The cells were cultured by rotating at 125 rpm at 37 °C and 8% CO_2_ for 5 days after transfecting the cells with 26 μg of the plasmid. The culture supernatant was collected via centrifugation for 10 min at 5000 × *g*, dialyzed with a solution of 20 mM Tris-HCl (pH 8.0) and 500 mM NaCl, 5 mM imidazole (binding buffer), and filtered through 0.8 μm filters (Advantec). It was loaded onto 1 mL of Ni-NTA Agarose resin (Qiagen, Germany) equilibrated with a binding buffer. After washing the resin with 10 mL binding buffer, the protein was eluted with buffers containing increasing concentrations of imidazole. The RBD was obtained after further purification using SEC with HiLoad 16/600 Superdex 75 pg column equilibrated with PBS at 4 °C.

### Circular Dichroism (CD) Spectroscopy

CD spectra of the h3C1 antibodies were recorded using a CD spectrometer (model J-1500, JASCO, Japan). Measurements were performed with 1.5 μM samples in 1× PBS using a 0.1 cm cell and a bandwidth of 1 nm. Five spectra were recorded for each sample.

### Differential Scanning Calorimetry (DSC)

The thermal stability was evaluated using a MicroCal PEAQ-DSC automated system (Malvern Panalytical, UK). Each IgG sample (1 mg/mL) in 1× PBS was loaded onto a 96-well plate (WEL190010-010, Malvern Panalytical) and placed in the sample compartment at 15 °C. Each sample–buffer pair was scanned over a range of 20–110 °C at a rate of 1 °C/min in each buffer. The measurements were repeated thrice in the same manner. The data were analyzed using the MicroCal PEAQ-DSC software version 1.40, and peak integration was performed using a nontwo-state model.

### Surface Plasmon Resonance (SPR)

The kinetic parameters of antigen–antibody binding were determined using SPR with a Biacore T200 instrument (Cytiva). h3C1 IgG was captured on the second flow cell of a protein A sensor chip (Cytiva) at approximately 300 resonance units. The RBD was injected into the sensor chip at a flow rate of 30 μL/min at 25 °C. The binding responses at the following concentrations of 3.13, 6.25, 12.5, 25, 50, and 100 nM of RBD were used for the experiment. The association and dissociation times were 100 and 800 s, respectively. The assays were performed in PBS-T buffer (1× PBS and 0.005% (v/v) Tween 20 surfactant). Biacore Insight Evaluation Software (Cytiva) was used to calculate the binding parameters. Measurements were repeated four times in the same manner.

### Quantification of Antibody Adsorption on NanoAct (before the Washing Step)

The amount of antibody adsorbed on NanoAct (cat. #RE1AA, Asahi Kasei, Japan) particles was quantified using a QuantiPro BCA protein assay kit (Sigma-Aldrich, USA). A mixture of 4.0 μg of antibody solution, 3.81 μL of the NanoAct suspension (1.05% (w/w)), and 34.2 μL of 10 mM phosphate buffer (pH 5.0, 7.0, and 9.0) was incubated at 37 °C for 2 h. The mixture was centrifuged at 17,900 × *g* for 20 min. Thirty μL of the supernatant was reacted with the BCA solution at 60 °C for 60 min. The absorbance of the purple solution was measured at 562 nm by using a plate reader (Infinite 200PRO M Nano, TECAN, Switzerland). The amount of antibody adsorbed on NanoAct was calculated using calibration curves obtained for each antibody.

### Quantification of Antibody Adsorption on NanoAct (after the Washing Step)

The amount of antibody adsorbed on NanoAct particles was quantified using a QuantiPro BCA protein assay. A mixture of 4.0 μg of antibody solution, 3.81 μL of the NanoAct suspension (1.05% (w/w)), and 34.2 μL of 10 mM phosphate buffer (pH 5.0, 7.0, and 9.0) was incubated at 37 °C for 2 h. For “pseudo” casein blocking, 457 μL of 100 mM borate buffer, pH 8.5, was added to the mixture and incubated at 37 °C for 60 min. The mixture was centrifuged at 17,900 × *g* for 20 min to obtain NanoAct precipitate, and then 457 μL of 50 mM borate buffer, pH 10.0, was added to the precipitate to thoroughly remove unbound antibodies. The mixture was centrifuged at 17,900 × *g* for 20 min, and then the precipitated antibody-bound NanoAct particles were dispersed in 80 μL of 1× D-PBS (−), which was prepared by diluting 10× D-PBS (−) (FUJIFILM Wako Pure Chemical, Japan) with distilled water to a final concentration of 0.05% (w/w), yielding the antibody-conjugated NanoAct dispersion. Then, the dispersion (8 μL) was reacted with the BCA solution at 60 °C for 60 min, and the mixture was centrifuged at 17,900 × *g* for 20 min. The absorbance of the purple supernatant was quantified at 450 nm using an Infinite 200PRO M Nano plate reader. The amount of antibody adsorbed on NanoAct was calculated using calibration curves obtained for each antibody.

### Quantification of Antibody Accessibility on NanoAct

Antibody-conjugated NanoAct dispersion was prepared following the same procedure as described above, except for the final step involving the resuspension of the conjugate particles. In this experiment, the precipitated antibody-bound NanoAct particles were resuspended in 80 μL of 1× D-PBS (−) and adjusted to a final concentration of 0.05% (w/w). Next, goat anti-human IgG polyclonal antibodies (Fab specific, cat. #I5260, RRID: AB_260206; Fc specific, cat. #I2136, RRID: AB_260147; Sigma-Aldrich) were labeled with horseradish peroxidase using a peroxidase-labeling kit (cat. #LK11, Dojindo, Japan). Then, a mixture of 8 μL of the antibody-conjugated NanoAct dispersion, 49 μL of 1× D-PBS (−), and 5.5 μL of 80 μg/mL HRP-labeled anti-human IgG polyclonal antibody was incubated at 37 °C for 1 h. The mixture was centrifuged at 17,900 × *g* for 20 min to obtain NanoAct precipitate, and then 300 μL of 1× D-PBS (−) was added to the precipitate to remove unbound HRP-labeled antibodies. After repeating this washing procedure four times, the precipitated antibody-bound NanoAct particles were resuspended in 40 μL of 1× D-PBS (−), adjusted to a final concentration of 0.01% (w/w), to obtain the antibody-conjugated NanoAct dispersion. Then, 1 μL of the dispersion was reacted with 10 μL of a 1:1 (v/v) mixture of 3,3′,5,5′-tetramethylbenzidine reagents (cat. # 5120-0048 and 5120-0037, Seracare, USA) at room temperature for 30 min in 96-well plates. One hundred microliters of 1 mol/L HCl aqueous solution was added to stop the reaction, and the absorbance at 450 nm was quantified using a plate reader (Infinite 200PRO M Nano). The amount of HRP-labeled antibody bound to NanoAct was calculated using calibration curves.

### Isothermal Titration Calorimetry (ITC)

Calorimetric titration of IgGs with NanoAct particles was performed on a MicroCal iTC200 (Malvern Panalytical) according to a previous study.^15^ Briefly, 10 mM phosphate buffer (at pH 5.0, 7.0, and 9.0) was added to the 1.06% (w/w) NanoAct suspension, centrifuged at 20,000 × *g* for 10 min to remove the supernatant, and then further suspended in the buffer. This procedure was repeated three times to produce a suspension with a final concentration of 0.2% (w/w) (0.13 nM). Then, 3.0 μL of the IgG solution (15 μM, or 25 μM of h3C1 IgG c−10/Fc-pos14 at pH 9.0) was titrated per injection 11 times at intervals of 120 s with a stirring rate of 750 rpm at 37 °C to the 0.13 nM NanoAct dispersion in the cell. The Fab and Fc domains were similarly measured at pH 9.0. Subsequently, 2.5 μL of the Fab or Fc solution was titrated per injection 14 times to the 65 pM NanoAct dispersion in the cell. Data were analyzed using the MicroCal Origin 7 software (Malvern Panalytical).

### Preparation of Antibody-Conjugated NanoAct Dispersion and Conjugate Pad

Preparation of antibody-conjugated NanoAct dispersion was conducted as described previously. NanoAct (the particle size is 341 nm) was used as the label nanoparticle in this study. For the conjugation of antibodies to NanoAct particles, 3.81 μL of 1.05% (w/w) NanoAct dispersion, 34.2 μL of 10 mM phosphate buffer solution (pH 5.0, 7.0, and 9.0), and 2.0 μL of 2 mg/mL (each) antibody solution were mixed and incubated at 37 °C for 120 min. For blocking, 457 μL of 1% (w/w) casein in 100 mM borate buffer (pH 8.5) was added to the mixture and incubated at 37 °C for 60 min. The mixture was centrifuged at 17,900 × *g* for 20 min to obtain NanoAct precipitate, and then 457 μL of 50 mM borate buffer (pH 10.0) was added to the precipitate to remove unbound antibodies and casein. The mixture was centrifuged at 17,900 × *g* for 20 min, and then the precipitated antibody-bound NanoAct particles were resuspended in 105 mg of suspension buffer (50 mM borate buffer (pH 10.0), 10% (w/w) trehalose, 4% (w/w) histidine, and 0.4% (w/w) casein), adjusted to a final concentration of 0.038% (w/w), to obtain antibody-conjugated NanoAct dispersion. A polyethylene conjugate pad (cat. #6613H, Ahlstrom, Finland) was cut into strips measuring 10 mm wide and 40 mm long. Following this, 106 μL of the 0.038% (w/w) NanoAct-antibody dispersion was evenly applied to the strip using a micropipette, and the strips were dried at 37 °C for 30 min (Figure S8).

### Preparation of Immunochromatography Test Strips

The immunochromatographic test strip consisted of a conjugate pad, a sample pad, a nitrocellulose membrane, and an absorbent pad. A cellulose sample pad (cat. #CBSP097, Asahi Kasei, Japan) was briefly immersed in a large excess of 66 mM phosphate buffer (pH 7.4), 0.9% (w/w) NaCl, 1.0% (w/w) BSA (Sigma-Aldrich), 6.0% (w/w) skim milk, and 2.0% (w/w) Tween 20 and was dried at 50 °C for 120 min. It was cut into strips 22 mm wide and 100 mm long. Unisart CN95 nitrocellulose membranes (Sartorius, Germany) were coated with antibody solutions using a SHOTMASTER 300DS liquid ejector (Musashi Engineering, Japan). Anti-SARS-CoV-2 Spike S2 Subunit monoclonal mouse IgG (cat. # MAB10557, RRID: AB_3657292, R&D Systems, USA) was used as a test line. Goat anti-human immunoglobulin (Fab specific, cat. #I5260, Sigma-Aldrich) was used as a control. The antibody solution for the test line was prepared at a concentration of 1 mg/mL in 10 mM phosphate buffer (pH 7.4) containing 0.9% (w/w) NaCl, 0.095% (w/w) NaN_3_, 2% (w/w) sucrose and 0.5% (w/w) BSA. The antibody solution for the control line was prepared at 0.5 mg/mL in 66 mM phosphate buffer (pH 7.4) containing 0.9% (w/w) NaCl. The antibody solution for the test line was sprayed at 1.0 μL per 1.0 cm with a width of 1 mm, positioned 9 mm from one end of the nitrocellulose membrane. Similarly, the antibody solution for the control line was sprayed under the same conditions, 15 mm from the same end. The nitrocellulose membranes were then dried at 50 °C for 24 h. Finally, the sample pad, the conjugate pad containing the NanoAct-labeled antibody, the antibody-coated nitrocellulose membrane, and the absorption pad (cat. #470, 25 mm wide, Whatman, UK) were attached to a 60 mm-long backing card (cat. #AR9020, Adhesives Research, USA) and subsequently cut into 3.8 mm-wide strips using a guillotine cutter (CM5000, BioDot, USA) to produce the LFIA test strips (Figure S9).

### Immunodetection of SARS-CoV-2 Spike Protein

The SARS-CoV-2 spike protein antigen (cat. #Z03481, GenScript, USA) was serially diluted to concentrations ranging from 3.13 to 100 ng/mL in application buffer (200 mM Tris-HCl, pH 8.0, 200 mM NaCl, 1.5% (w/w) Triton X-100, and 0.5% (w/w) Tween 20) to prepare positive specimens. Each immunochromatography test strip was placed in a plastic housing, and an aliquot of 80 μL of the positive specimens was added to the sample compartment. After a 20 min incubation at room temperature, the color intensities of both the test and control lines were measured using a TOR-500 (Trustmedical, Japan) and analyzed using TMreader image analysis software (ver.1.3.0.5, Trustmedical). To quantify false-positive signals, 80 μL of application buffer without antigen was also tested under the same conditions. For each condition, three test strips were analyzed to determine the average color intensity. A threshold value of 3.0 was set for the optical reader to distinguish between positive and negative results (Figures 5 and S10).

## Supplementary Material



## Data Availability

The authors have cited additional references in the Supporting Information.^30^

## Notes

Physicochemical properties of h3C1 IgGs, schematic illustrations of the experimental procedures, detailed comparative analysis of immunodetection sensitivity, and binding stoichiometry of antibody onto NanoAct (PDF)
