# Supplementary material for: Hybrid Supercharged Antibodies: A Rational Approach to Boost Immunoassay Sensitivity via Controlled Nanoparticle Adsorption
Source: Langmuir. 2026 Apr 3;42(15):10303–13. doi: 10.1021/acs.langmuir.5c06029 (PMC13105228; doi:10.1021/acs.langmuir.5c06029)
Supplement: Supplementary file 1 [file la5c06029_si_001.pdf]

Supporting Information for

Hybrid Supercharged Antibodies: A Rational Approach to Boost Immunoassay Sensitivity via Controlled Nanoparticle Adsorption

(Authors)

Junichi Sato [1,2‡], Keisuke Kasahara [3,4‡], Satoru Nagatoishi [3,5], Keisuke Murakami [2], Daisuke Kuroda [6], Jose M M Caaveiro [4], Hirokazu Nagai [2], Kouhei Tsumoto [1,3,5*]

(Affiliations)

[1] Department of Chemistry and Biotechnology, Graduate School of Engineering, The University of Tokyo, 7-3-1, Hongo, Bunkyo-ku, Tokyo 113-8656, Japan.

[2] Biomaterial Business Development Department, Asahi Kasei Corporation, Hibiya Mitsui Tower 1-1-2 Yurakucho, Chiyoda-ku, Tokyo 100-0006, Japan.

[3] Department of Bioengineering, Graduate School of Engineering, The University of Tokyo, 7-3-1, Hongo, Bunkyo-ku, Tokyo 113-8656, Japan.

[4] Laboratory of Protein Drug Discovery, Graduate School of Pharmaceutical Sciences, Kyushu University, 3-1-1 Maidashi, Higashi-ku, Fukuoka 812-8582, Japan.

[5] Medical Device Development and Regulation Research Center, School of Engineering, The University of Tokyo, 7-3-1 Hongo, Bunkyo-ku, Tokyo 113-8656, Japan.

[6] Department of Biosciences, College of Humanities and Sciences, Nihon University, 3-25-40 Sakurajosui Setagaya-ku, Tokyo 156-8550, Japan.

‡ These authors contributed equally to this work.

*Corresponding Author: Kouhei Tsumoto (tsumoto@bioeng.t.u-tokyo.ac.jp)

Supplementary Tables

Table S1. Isoelectric point of the Fab and Fc domains of h3C1 IgGs. Theoretical pI was calculated using PDB2PQR [16–18].

	pI (Theoretical)
WT	Fab: 9.0 / Fc 7.6
Fc-pos14	Fab: 9.0 / Fc 9.7
c−10	Fab: 6.5 / Fc 7.6
c−10/Fc-pos14	Fab: 6.5 / Fc 9.7

Table S2. Thermal stability of h3C1 IgGs. Values are mean ± S.D. of three independent experiments.

	T_m1 (°C)	T_m2 (°C)	$\Delta H1$ (kcal/mol)	$\Delta H2$ (kcal/mol)
WT	72.5 ± 0.0	82.8 ± 0.0	938 ± 6	218 ± 3
Fc-pos14	72.5 ± 0.0	83.1 ± 0.0	940 ± 11	203 ± 6
c−10	71.6 ± 0.0	82.4 ± 0.0	875 ± 8	201 ± 13
c−10/Fc-pos14	71.5 ± 0.0	82.2 ± 0.0	912 ± 13	210 ± 13

Table S3. Kinetic parameters of the interaction between h3C1 IgGs and the antigen RBD. Values are mean ± S.D. of four independent experiments.

	k_{on} (10^5 $M^{-1}\cdot s^{-1}$)	k_{off} (10^{-4} s^{-1})	K_D (pM)
WT	7.54 ± 0.14	5.68 ± 0.06	753 ± 20
Fc-pos14	6.91 ± 0.18	5.49 ± 0.06	795 ± 18
c−10	8.14 ± 0.26	4.71 ± 0.06	579 ± 25
c−10/Fc-pos14	7.66 ± 0.22	4.59 ± 0.09	599 ± 23

Table S4. Thermodynamic parameters of the interaction between NanoAct™ particles and h3C1 IgGs at pH 9.0. Values are mean ± S.D. of three independent experiments.

		ΔG (kcal/mol)	ΔH (kcal/mol)	$-T\Delta S$ (kcal/mol)
IgG	WT	−10.1 ± 0.2	−105.7 ± 5.9	95.6 ± 6.1
	Fc-pos14	−10.0 ± 0.2	−70.6 ± 7.1	60.7 ± 7.0
	c−10	N.D.*	N.D.*	N.D.*
	c−10/Fc-pos14	−10.8 ± 0.0	−51.3 ± 0.3	40.5 ± 0.3
Fab	WT	−8.3 ± 0.4	N.D.**	N.D.**
	c−10	N.D.*	N.D.*	N.D.*
Fc	WT	N.D.*	N.D.*	N.D.*
	pos14	−10.9 ± 0.5	−19.0 ± 1.1	−8.0 ± 1.6

*Not determined. The reaction curve could not be obtained.

**Not determined. The affinity was weak, and the N value was inaccurate. From that N value, thermodynamic parameters were also unreliable.

Table S5. Thermodynamic parameters of the interaction between NanoAct™ particles and h3C1 IgGs at pH 5.0.

	N	N_{sup}	K_D (nM)	ΔG (kcal/mol)	ΔH (kcal/mol)	$-T\Delta S$ (kcal/mol)
WT	4500	>20500	N.D.*	N.D.*	N.D.*	N.D.*
Fc-pos14	5000	>20500	N.D.*	N.D.*	N.D.*	N.D.*
c−10	8000	>20500	N.D.*	N.D.*	N.D.*	N.D.*
c−10/Fc-pos14	6300	>20500	N.D.*	N.D.*	N.D.*	N.D.*

*Not determined. The endothermic reactions were small and analyzed with low accuracy.

Table S6. Thermodynamic parameters of the interaction between NanoAct™ particles and h3C1 IgGs at pH 7.0. Values are mean ± S.D. of two or three independent experiments.

	N	N_{sup}	K_D (nM)	ΔG (kcal/mol)	ΔH (kcal/mol)	$-T\Delta S$ (kcal/mol)
WT	11200 ± 600	>20500	25.4 ± 7.5	−10.4 ± 0.2	−94.9 ± 6.3	84.6 ± 6.5
Fc-pos14	8700 ± 2000	>20500	112.2 ± 45.4	−9.5 ± 0.3	−91.7 ± 3.0	82.2 ± 3.1
c−10	5900 ± 700	15700 ± 1000	26.9 ± 8.7	−10.4 ± 0.2	−108.6 ± 8.7	98.2 ± 8.9
c−10/Fc-pos14	11400 ± 200	>20500	273.6 ± 255.9	−9.1 ± 0.7	−80.3 ± 28.6	71.1 ± 29.3

Supplementary Figures

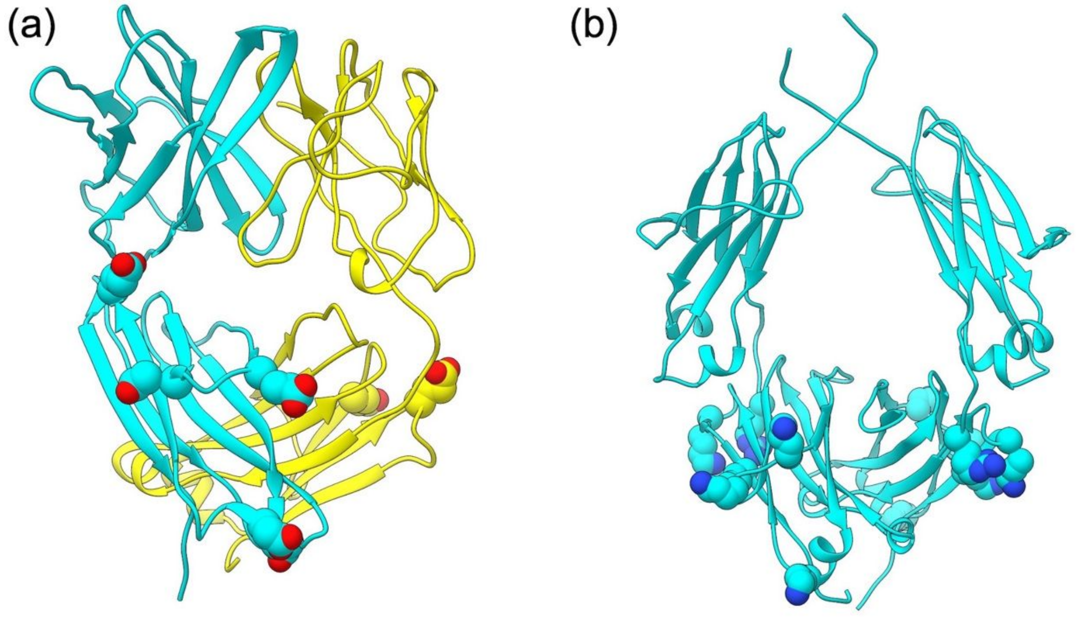

(c) human IgG1 C_H1

```
              118                                                                                                          220
WT            AST KGPSVFPLAP SSKSTSGGTA ALGCLVKDYF PEPVTVSWNS GALTSGVHTF PAVLQSSGLY SLSSVVTVPS SSLGTQTYIC NVNHKPSNTK VDKKVEPKSC
c-10 (-7)     AST KGPSVFPLAP SSDSTSGGTA ALGCLVKDYF PEPVTVSWND GALTEGVHTF PAVLQSSGLY SLSSVVTVPS DSLGTQTYIC NVNHEPSNTK VDKKVEPKSC
```

(d) human IgGκ C_L

```
              108                                          150                                                                200        214
WT            RTV AAPSVFIFPP SDEQLKSGTA SVVCLLNNFY PREAKVQWKV DNALQSGNSQ ESVTEQDSKD STYSLSSTLT LSKADYEKHK VYACEVTHQG LSSPVTKSFN RGEC
c-10 (-3)     RTV AAPSVFIFPP SDEQLKSGTA SVVCLLNNFY PREAEVQWKV DNALQSGNSQ ESVTEQDSKD STYSLSSTLT LSKADYEKHK VYACEVTHEG LSSPVTKSFN RGEC
```

(e) human IgG1 Fc

```
              342  350                                                    400                                               443
pos14 (+7)    QPRRPQVYT LPPSRDELTK NQVSLTCLVK GFYPSDIAVE WESNGKPEKN YKTTPPVLDS DGSFFLYSKL TVDKSRWQKG NVFSCSVMHE ALKNHYTKKS LSL
WT            QPREPQVYT LPPSRDELTK NQVSLTCLVK GFYPSDIAVE WESNGQPENN YKTTPPVLDS DGSFFLYSKL TVDKSRWQQG NVFSCSVMHE ALHNHYTQKS LSL
```

Figure S1. Computational design of supercharged IgGs. Structures of (a) h3C1 Fab c−10 mutant and (b) human IgG1 Fc-pos14 mutant. The heavy and light chains are indicated in cyan and yellow, respectively. The designed charged residues are shown as spheres. Amino acid sequences of (c) C_H1 and (d) C_L of h3C1 Fab and (e) C_H3 of Fc (EU numbering scheme).

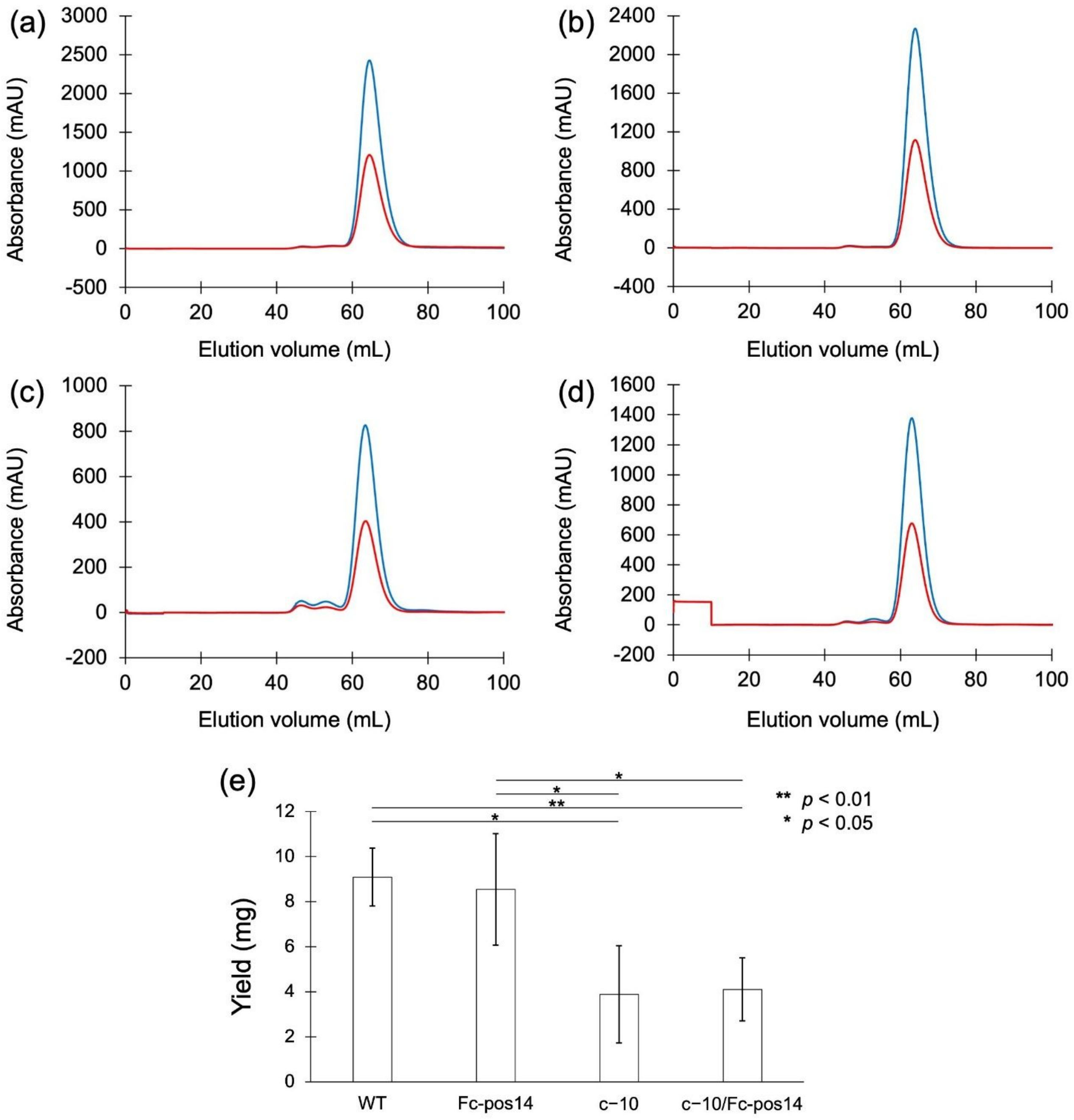

Figure S2. Size-exclusion chromatography and expression levels of h3C1 IgGs. SEC profiles of (a) WT, (b) Fc-pos14, (c) c − 10, and (d) c − 10/Fc-pos14. Representative examples from multiple datasets are shown. Absorbance at 280 and 260 nm is shown as blue and red lines, respectively. The optical path length was 1 cm. (e) Comparison of yields obtained from a 25 mL cell culture.

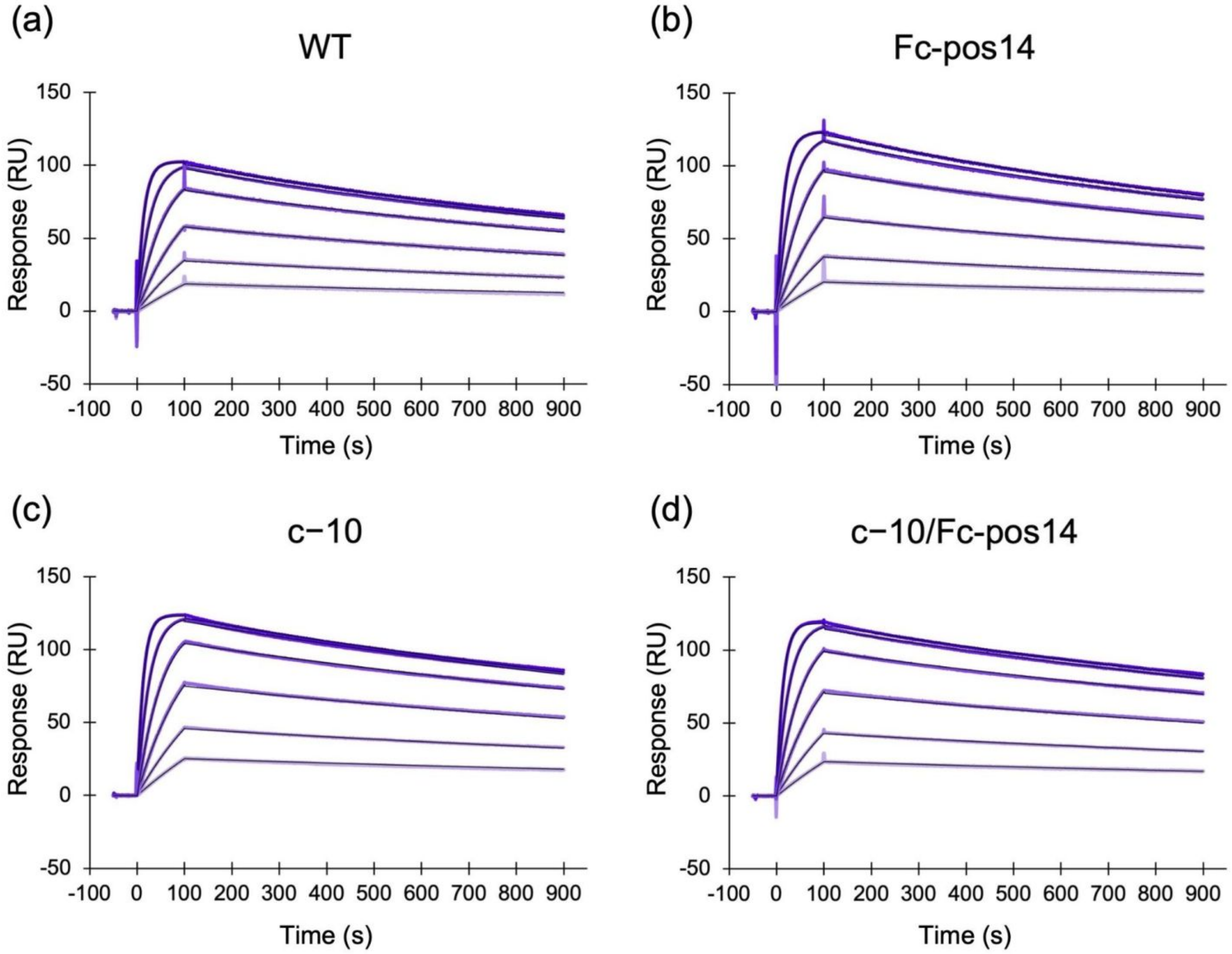

Figure S3. SPR response vs. time curves for interaction of RBD with h3C1 IgG. (a) WT, (b) Fc-pos14, (c) c−10, and (d) c−10/Fc-pos14 at 25 °C. The raw sensorgrams are shown as purple lines, with the color intensity indicating analyte concentration: the darkest line corresponds to the highest concentration (100 nM). The curve fitting profiles are shown as black lines. K_D values (mean ± S.D., n = 4): 753 ± 20 pM (WT), 795 ± 18 pM (Fc-pos14), 579 ± 25 pM (c−10), and 599 ± 23 pM (c−10/Fc-pos14).

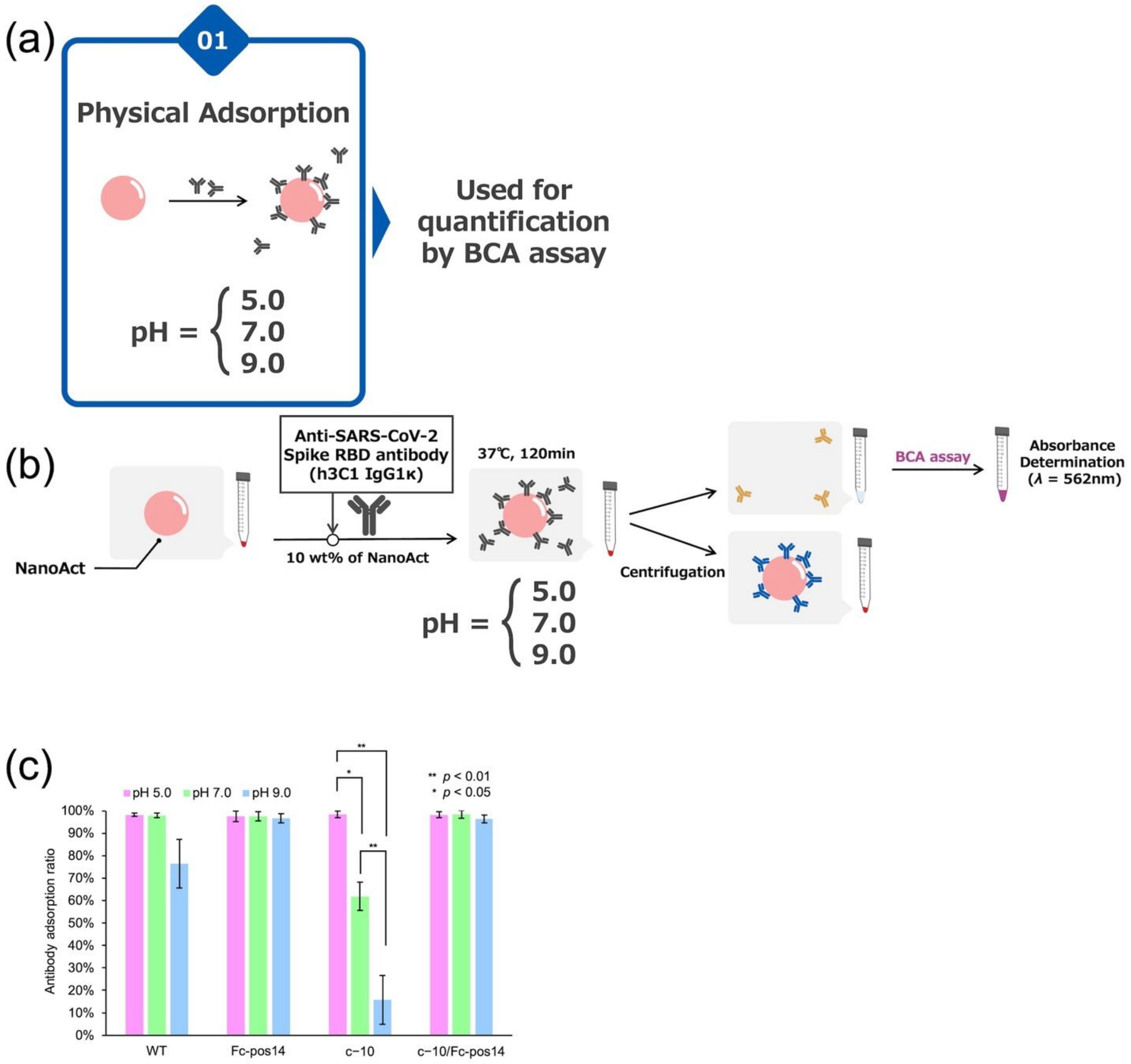

Figure S4. Quantification of antibody adsorption on NanoAct™ before the washing step. (a) A diagrammatic explanation of the quantitative method focusing on the physical adsorption step. (b) Detailed explanation of the quantitative method for antibody adsorption. (c) The antibody adsorption ratio measured before the washing step in the conjugation process. The amount of antibody fed in the physical adsorption step is defined as 100%. A significance test was conducted between different pH conditions for each antibody but not conducted between different antibodies.

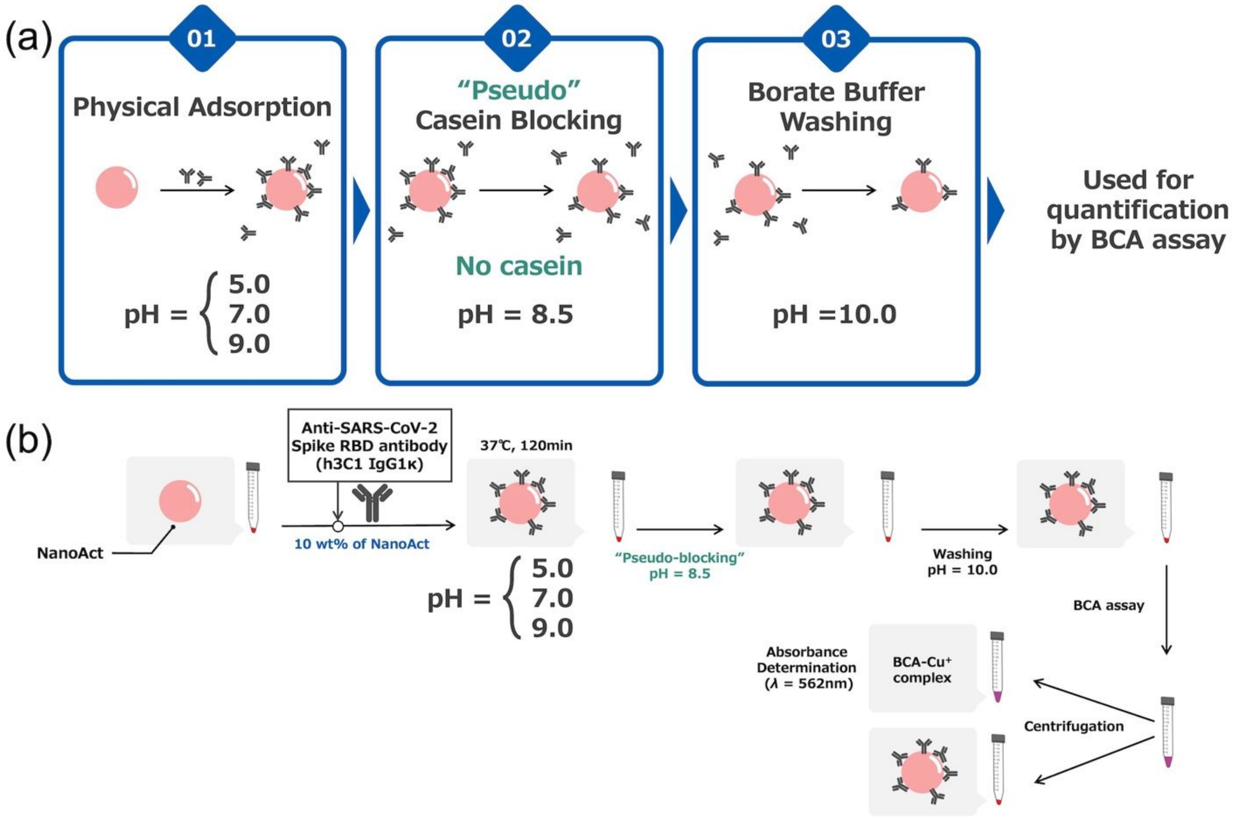

Figure S5. Quantification of antibody adsorption on NanoAct^{TM} after the washing step. (a) A diagrammatic explanation of the quantitative method focusing on the conjugation process. There are three main steps in the conjugation process: (1) physical adsorption, (2) casein blocking, and (3) borate buffer washing. Since casein interferes with the BCA assay, a borate buffer without casein was employed in step 2 in this experiment. (b) Detailed explanation of the quantitative method for antibody adsorption.

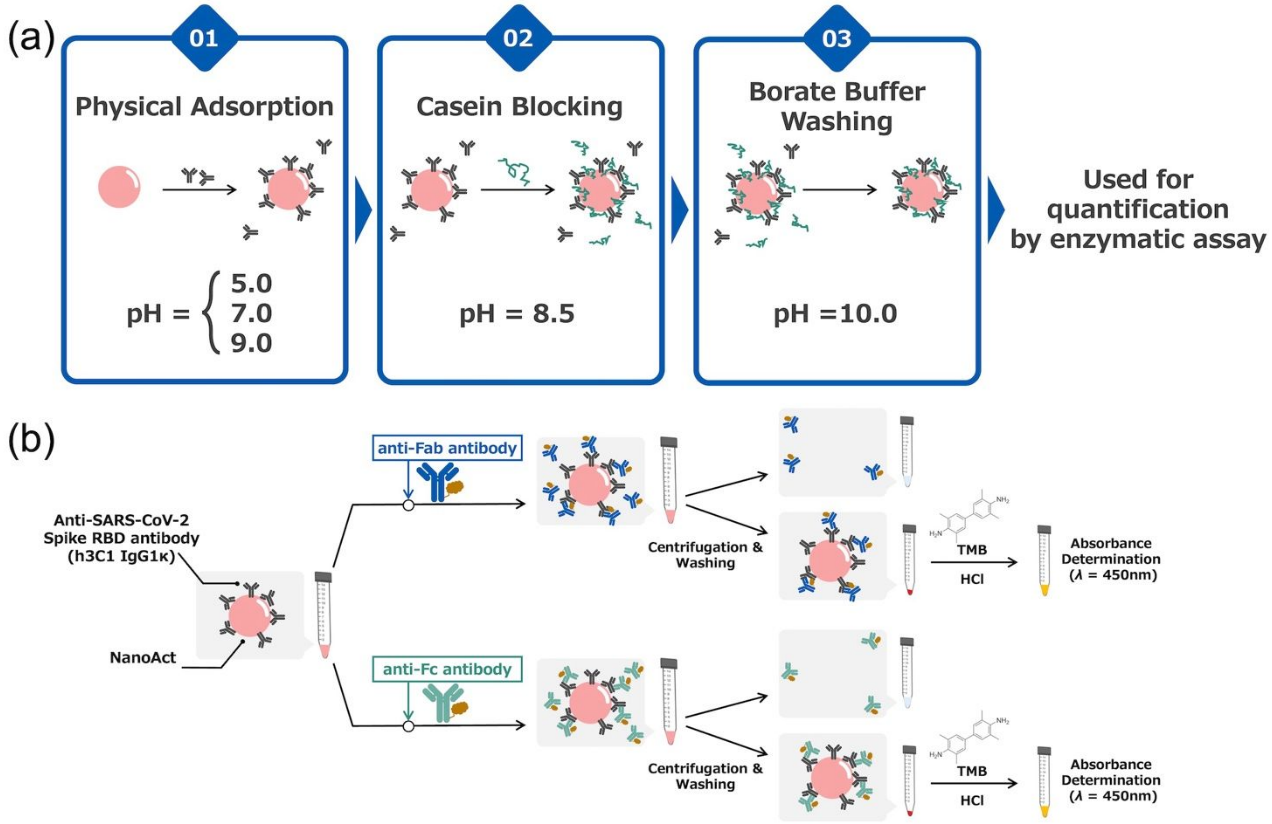

Figure S6. Quantification of antibody accessibility on NanoAct™. (a) A diagrammatic explanation of the quantitative method focusing on the three steps in the conjugation process. Since casein does not interfere with the enzymatic assay, a borate buffer with casein was employed in step 2 in this experiment. (b) Detailed explanation of the quantitative method for Fab and Fc accessibility.

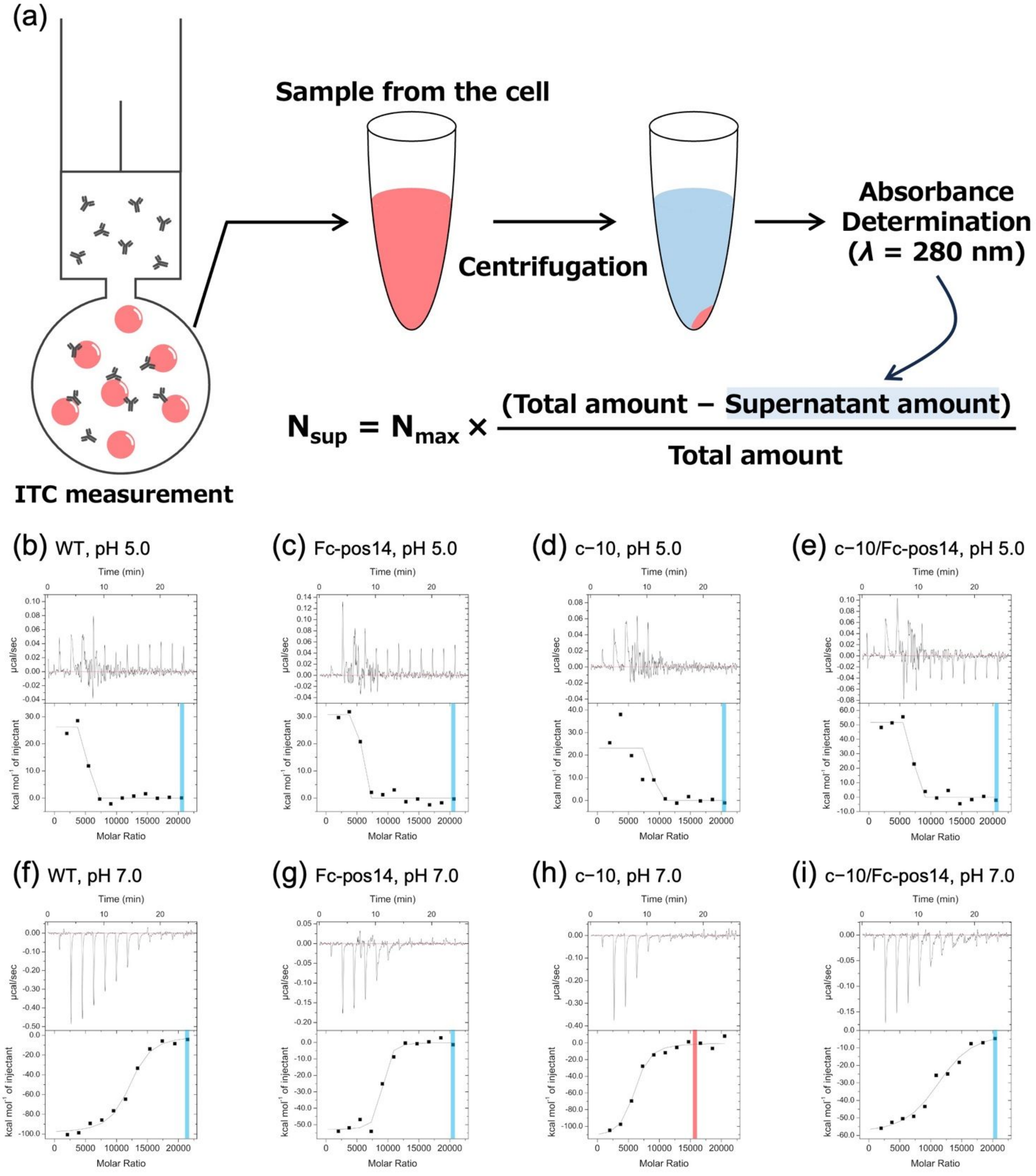

Figure S7. ITC analyses of the interaction between NanoAct™ particles and IgGs. (a) How to calculate N_{sup} values after ITC measurements. (b–e) WT and mutants (Fc-pos14, c−10, c−10/Fc-pos14) at pH 5.0; (f–i) WT and mutants at pH 7.0. Red or blue lines indicate the average N_{sup} in the measurements, with red representing unsaturated and blue representing saturated conditions.

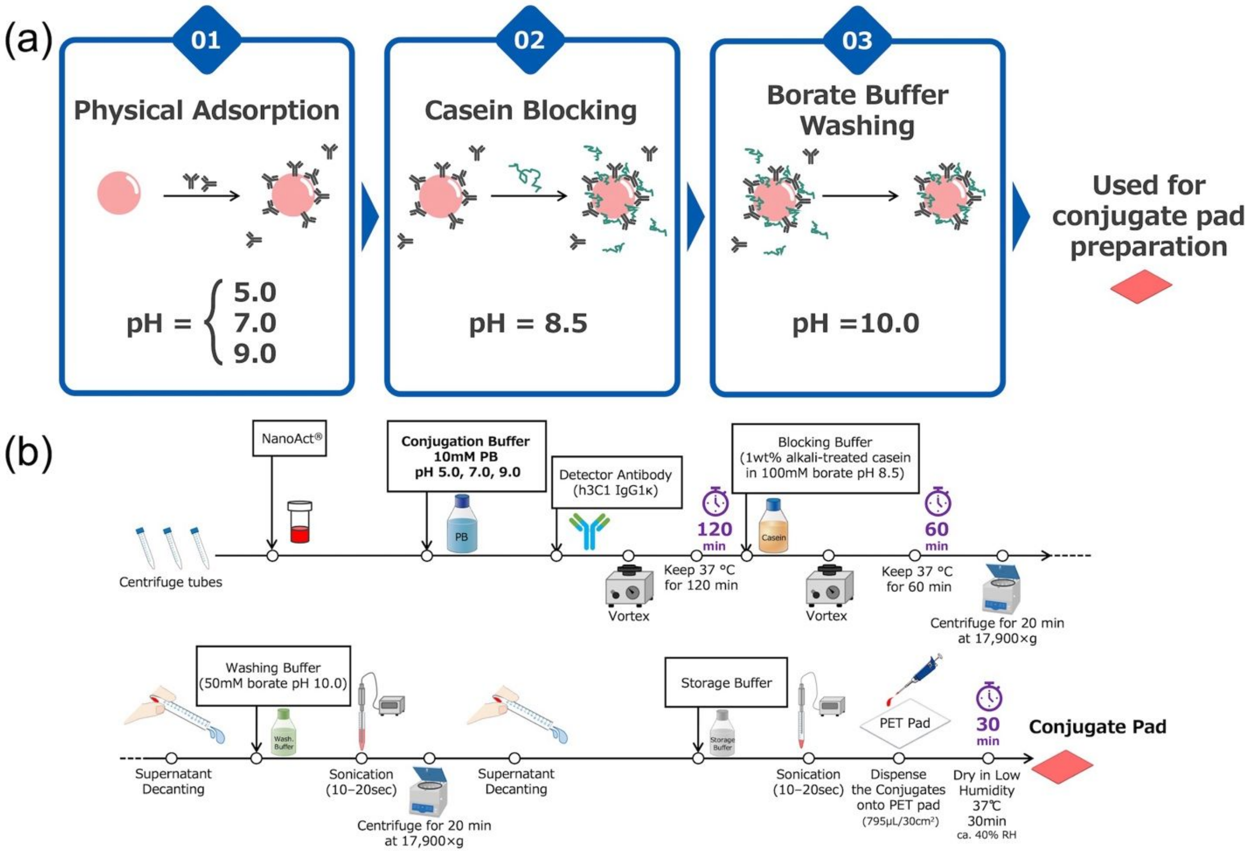

Figure S8. Preparation of antibody-conjugated NanoAct™ dispersion and conjugate pad. (a) A diagrammatic explanation of the preparation method for conjugate pads focusing on the three steps in the conjugation process. (b) Detailed explanation of the preparation method for conjugate pads.

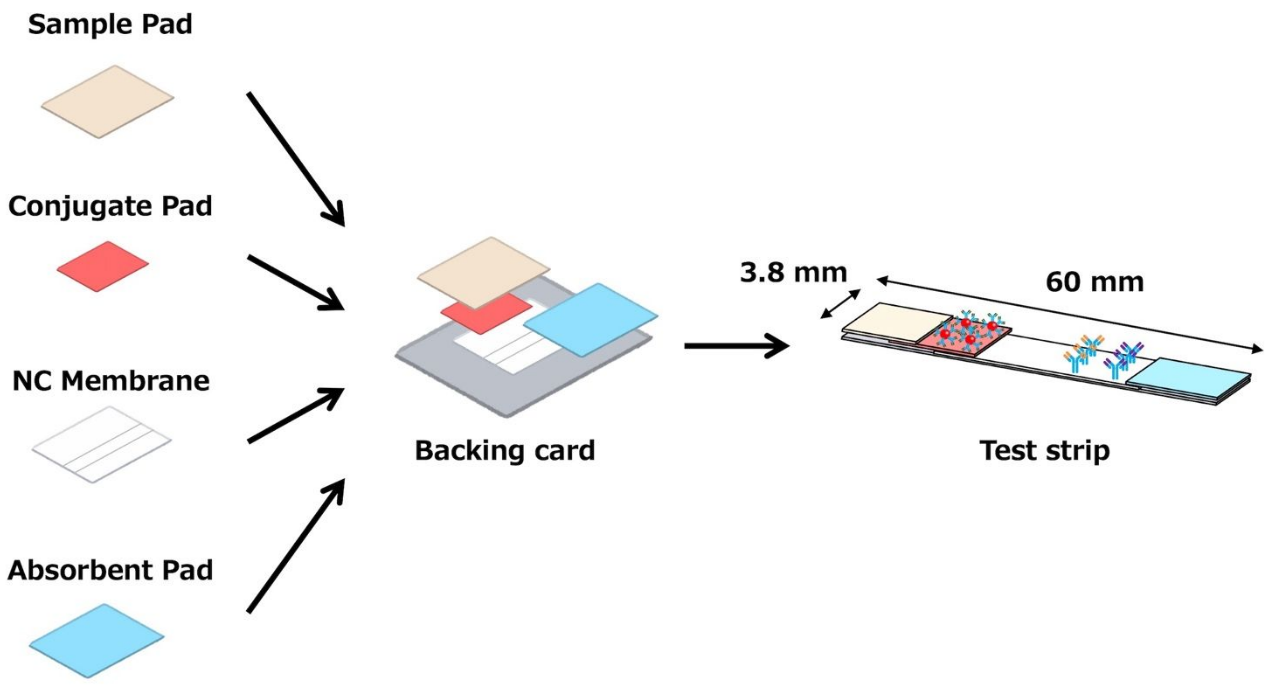

Figure S9. Preparation scheme of LFIA test strips.

(a)

Antibody		WT			Fc-pos14			c−10			c−10/Fc-pos14		
pH of conjugation step		5.0	7.0	9.0	5.0	7.0	9.0	5.0	7.0	9.0	5.0	7.0	9.0
Antigen Conc. (ng/mL)	100	(10.5)	(9.9)	(7.7)	(12.9)	(15.4)	(24.2)	(11.6)	(8.8)	(2.2)	(18.9)	(15.5)	(25.4)
	50	5.9	6.0	3.3	(8.1)	(8.9)	(17.4)	6.9	5.6	1.9	(16.4)	(9.2)	(19.5)
	25	3.3	3.4	2.9	4.6	5.2	10.5	4.5	3.2	0.9	8.6	5.4	11.9
	12.5	2.6	2.4	2.1	3.0	3.3	6.3	2.7	2.0	1.3	5.8	3.5	7.8
	6.25	2.1	1.8	1.0	2.1	2.0	4.3	1.9	1.3	(0.0)	3.6	2.3	4.5
	3.13	(1.2)	(1.0)	(1.4)	1.7	1.4	2.9	N.A.	N.A.	N.A.	2.6	1.9	3.4
	0	0.4	1.3	0.4	0.4	1.1	1.4	1.2	0.9	0.9	1.9	1.4	0.5

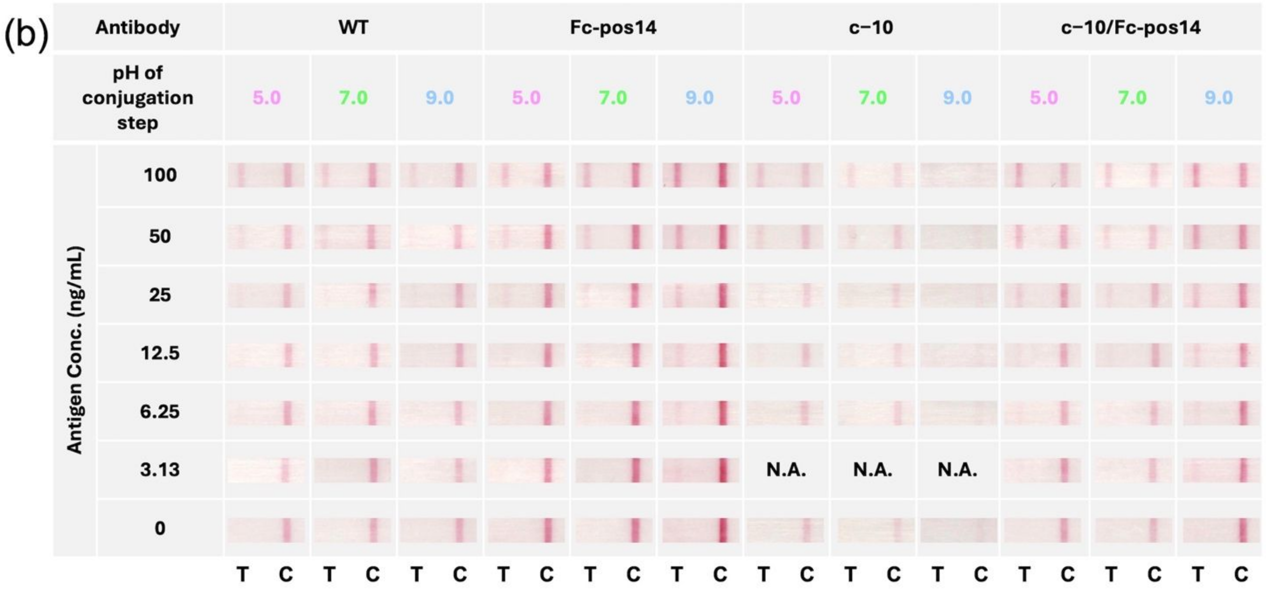

Figure S10. Detailed comparative analysis of immunodetection sensitivity for SARS-CoV-2 spike protein across varying antigen gradients and antibody adsorption conditions. (a) Average color intensity of the test line for LFIA devices using NanoAct™ conjugated with each antibody at different pH levels. The values in parentheses represent the results obtained from a single measurement. (b) Photographs of LFIA test strips after immunodetection. "T" and "C" in the figure represent the positions of the test line and the control line, respectively.

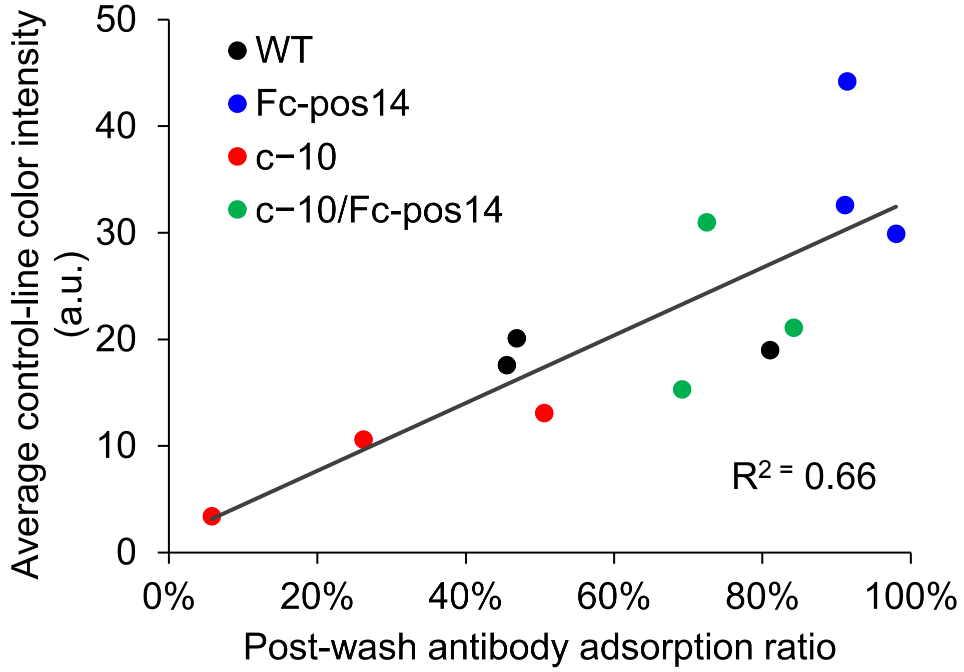

Figure S11. Correlation between antibody adsorption and control-line color intensity. Post-wash antibody adsorption ratios (Fig. 3a) and the corresponding average control-line color intensities of LFIA test strips (Fig. S10b) were plotted. Linear regression using all data points yielded a coefficient of determination (R^2) of 0.66.

Experimental Details

Binding stoichiometry of antibody onto NanoAct™

The theoretical maximum number of IgG molecules that could form a monolayer on a particle surface (r_{theor}) was calculated as follows: According to dynamic light scattering measurements, the average particle diameter was 341 nm, as reported in a previous study [15]. Assuming NanoAct™ is a perfect sphere with a smooth surface, the surface area of a single particle is 3.65×10^5 nm^2. Based on the literature [30], the dimensions of the IgG antibody measured using cryo-atomic force microscopy were approximately 12 nm (length) × 12 nm (width) × 3 nm (height). Accordingly, the minimum contact area (projected area) of an IgG molecule is calculated to be approximately 36 nm^2, and r_{theor} is estimated to be approximately 1.0×10^4.

The ratio of the antibody molecules to particles (r_{exp}) was calculated as follows: In the conjugation process, 4.0 μg of supercharged antibody was added to 3.81 μL of 1.05% (w/w) NanoAct™ suspension. Given that the density of the original NanoAct™ suspension was 1.00 g cm^{-3}, the mass of NanoAct™ added to the antibody was 40 μg. According to the previous study [15], the density of a standard NanoAct™ particle is assumed to be 1.41 g/cm^3, and its volume, calculated from an average diameter of 341 nm, is approximately 2.08×10^{-14} cm^{-3}. Therefore, the mass of a single particle is calculated to be 2.93×10^{-14} g. Considering that the typical molecular weight of IgG is 1.5×10^5 and that the Avogadro constant is 6.02×10^{23} mol^{-1}, r_{exp} was calculated using the following equation.

$$r_{exp} = \frac{(4.0 \times 10^{-6}) \div (1.5 \times 10^5) \times (6.02 \times 10^{23})}{(40 \times 10^{-6}) \div (2.93 \times 10^{-14})} = 1.2 \times 10^4$$

Abbreviations

LFIA, lateral flow immunoassay

pI, isoelectric point

WT, wild-type

T_m, midpoint temperature of denaturation

S.D., standard deviation

RBD, receptor binding domain

CHO, Chinese hamster ovary

SEC, size-exclusion chromatography

CD, circular dichroism

SPR, surface resonance plasmon

DSC, differential scanning calorimetry

PBS, phosphate-buffered saline

HRP, horseradish peroxidase

BCA, bicinchoninic acid assay

N.C., negative control

ITC, isothermal titration calorimetry

LOD, limit of detection

TL, the test line

CL, the control line

IgG, Immunoglobulin G

Fv, Fragment variable of antibody

Fc, Fragment crystallizable of antibody

Fab, Fragment antigen-binding of antibody

References

[1] J. Budd, B. S. Miller, N. E. Weckman, D. Cherkaoui, D. Huang, A. T. Decruz, N. Fongwen, G.-R. Han, M. Broto, C. S. Estcourt, J. Gibbs, D. Pillay, P. Sonnenberg, R. Meurant, M. R. Thomas, N. Keegan, M. M. Stevens, E. Nastouli, E. J. Topol, A. M. Johnson, M. Shahmanesh, A. Ozcan, J. J. Collins, M. Fernandez Suarez, B. Rodriguez, R. W. Peeling, R. A. McKendry, *Nat. Rev. Bioeng.* **2023**, *1*, 13–31.

[2] A. Sena-Torralba, R. Álvarez-Diduk, C. Parolo, A. Piper, A. Merkoçi, *Chem. Rev.* **2022**, *122*, 14881–14910.

[3] J. Kim, M.-S. Shin, J. Shin, H.-M. Kim, X.-H. Pham, S.-M. Park, D.-E. Kim, Y. J. Kim, B.-H. Jun, *Int. J. Mol. Sci.* **2023**, *24*, 9600.

[4] S. Gao, L. Niu, R. Zhou, C. Wang, X. Zheng, D. Zhang, X. Huang, Z. Guo, X. Zou, *Int. J. Biol. Macromol.* **2024**, *257*, 128621.

[5] A.-C. Mirica, D. Stan, I.-C. Chelcea, C. M. Mihailescu, A. Ofiteru, L.-A. Bocancia-Mateescu, *Front. Bioeng. Biotechnol.* **2022**, *10*, DOI 10.3389/fbioe.2022.922772.

[6] S. Gao, J. M. Guisán, J. Rocha-Martin, *Anal. Chim. Acta* **2022**, *1189*, 338907.

[7] G. Ruiz, K. Tripathi, S. Okyem, J. D. Driskell, *Bioconjug. Chem.* **2019**, *30*, 1182–1191.

[8] F. Kang, Y. Yang, J. Li, E. Chen, T. Hong, L. Zhao, M. Du, *Foods* **2022**, *11*, 3599.

[9] D. Lou, L. Ji, L. Fan, Y. Ji, N. Gu, Y. Zhang, *Langmuir* **2019**, *35*, 4860–4867.

[10] M. S. Lawrence, K. J. Phillips, D. R. Liu, *J. Am. Chem. Soc.* **2007**, *129*, 10110–10112.

[11] A. E. Miklos, C. Kluwe, B. S. Der, S. Pai, A. Sircar, R. A. Hughes, M. Berrondo, J. Xu, V. Codrea, P. E. Buckley, A. M. Calm, H. S. Welsh, C. R. Warner, M. A. Zacharko, J. P. Carney, J. J. Gray, G. Georgiou, B. Kuhlman, A. D. Ellington, *Chem. Biol.* **2012**, *19*, 449–455.

[12] B. S. Der, C. Kluwe, A. E. Miklos, R. Jacak, S. Lyskov, J. J. Gray, G. Georgiou, A. D. Ellington, B. Kuhlman, *PLoS One* **2013**, *8*, e64363.

[13] K. Kasahara, D. Kuroda, A. Tanabe, R. Kawade, S. Nagatoishi, K. Tsumoto, *Biochem. Biophys. Res. Commun.* **2021**, *563*, 54–59.

[14] K. Kasahara, M. Nakakido, D. Kuroda, S. Nagatoishi, K. Tsumoto, *Polym. J.* **2025**, *57*, 923–930.

[15] K. Murakami, S. Nagatoishi, K. Kasahara, H. Nagai, Y. Sasajima, R. Sasaki, K. Tsumoto, *Anal. Biochem.* **2021**, *632*, 114337.

[16] T. J. Dolinsky, J. E. Nielsen, J. A. McCammon, N. A. Baker, *Nucleic Acids Res.* **2004**, *32*, W665-7.

[17] T. J. Dolinsky, P. Czodrowski, H. Li, J. E. Nielsen, J. H. Jensen, G. Klebe, N. A. Baker, *Nucleic Acids Res.* **2007**, *35*, W522-5.

[18] S. Unni, Y. Huang, R. M. Hanson, M. Tobias, S. Krishnan, W. W. Li, J. E. Nielsen, N. A. Baker, *J. Comput. Chem.* **2011**, *32*, 1488–1491.

[19] C. Zhang, Y. Wang, Y. Zhu, C. Liu, C. Gu, S. Xu, Y. Wang, Y. Zhou, Y. Wang, W. Han, X. Hong, Y. Yang, X. Zhang, T. Wang, C. Xu, Q. Hong, S. Wang, Q. Zhao, W. Qiao, J. Zang, L. Kong, F. Wang, H. Wang, D. Qu, D. Lavillette, H. Tang, Q. Deng, Y. Xie, Y. Cong, Z. Huang, *Nat. Commun.* **2021**, *12*, 264.

[20] M. Kiyoshi, J. M. M. Caaveiro, T. Kawai, S. Tashiro, T. Ide, Y. Asaoka, K. Hatayama, K. Tsumoto, *Nat. Commun.* **2015**, *6*, 6866.

[21] J. U. Lee, W. Shin, J. Y. Son, K.-Y. Yoo, Y.-S. Heo, *Int. J. Mol. Sci.* **2017**, *18*, 228.

[22] E. Jurrus, D. Engel, K. Star, K. Monson, J. Brandi, L. E. Felberg, D. H. Brookes, L. Wilson, J. Chen, K. Liles, M. Chun, P. Li, D. W. Gohara, T. Dolinsky, R. Konecny, D. R. Koes, J. E. Nielsen, T. Head-Gordon, W. Geng, R. Krasny, G.-W. Wei, M. J. Holst, J. A. McCammon, N. A. Baker, *Protein Sci.* **2018**, *27*, 112–128.

[23] A. Sali, T. L. Blundell, *J. Mol. Biol.* **1993**, *234*, 779–815.

[24] C. Parolo, A. de la Escosura-Muñiz, E. Polo, V. Grazú, J. M. de la Fuente, A. Merkoçi, *ACS Appl. Mater. Interfaces* **2013**, *5*, 10753–10759.

[25] N. G. Welch, J. A. Scoble, B. W. Muir, P. J. Pigram, *Biointerphases* **2017**, *12*, 02D301.

[26] Y. Liu, L. Zhan, Z. Qin, J. Sackrison, J. C. Bischof, *ACS Nano* **2021**, *15*, 3593–3611.

[27] H. Park, P. Bradley, P. Greisen Jr, Y. Liu, V. K. Mulligan, D. E. Kim, D. Baker, F. DiMaio, *J. Chem. Theory Comput.* **2016**, *12*, 6201–6212.

[28] R. F. Alford, A. Leaver-Fay, J. R. Jeliazkov, M. J. O'Meara, F. P. DiMaio, H. Park, M. V. Shapovalov, P. D. Renfrew, V. K. Mulligan, K. Kappel, J. W. Labonte, M. S. Pacella, R. Bonneau, P. Bradley, R. L. Dunbrack Jr, R. Das, D. Baker, B. Kuhlman, T. Kortemme, J. J. Gray, *J. Chem. Theory Comput.* **2017**, *13*, 3031–3048.

[29] E. F. Pettersen, T. D. Goddard, C. C. Huang, G. S. Couch, D. M. Greenblatt, E. C. Meng, T. E. Ferrin, *J. Comput. Chem.* **2004**, *25*, 1605–1612.

[30] W. Han, J. Mou, J. Sheng, J. Yang, Z. Shao, *Biochemistry* **1995**, *34*, 8215–8220.
